# *Citrus* Extract as a Perspective for the Control of Dyslipidemia: A Systematic Review With Meta-Analysis From Animal Models to Human Studies

**DOI:** 10.3389/fphar.2022.822678

**Published:** 2022-02-14

**Authors:** Betina M. R. Carvalho, Laranda C. Nascimento, Jessica C. Nascimento, Vitória S. dos S. Gonçalves, Patricia K. Ziegelmann, Débora S. Tavares, Adriana G. Guimarães

**Affiliations:** ^1^ Programa de Pós-Graduação em Ciências Aplicadas à Saúde, Universidade Federal de Sergipe, Lagarto, Brazil; ^2^ Departamento de Química, Universidade Federal de Sergipe, São Cristóvão, Brazil; ^3^ Departamento de Estatística, Programa de Pós-graduação em Epidemiologia, Universidade Federal do Rio Grande do Sul, Porto Alegre, Brazil; ^4^ Departamento de Educação em Saúde, Universidade Federal de Sergipe, Lagarto, Brazil; ^5^ Departamento de Farmácia, Universidade Federal de Sergipe, São Cristóvão, Brazil

**Keywords:** dyslipidemia, citrus, hyperlipidemia, flavonoids, cholesterol

## Abstract

This study aims to obtain scientific evidence on the use of *Citrus* to control dyslipidemia. The surveys were carried out in 2020 and updated in March 2021, in the PubMed, Scopus, LILACS, and SciELO databases, using the following descriptors: *Citrus*, dyslipidemias, hypercholesterolemia, hyperlipidemias, lipoproteins, and cholesterol. The risk of bias was assessed according to the Cochrane methodology for clinical trials and ARRIVE for preclinical trials. A meta-analysis was performed using the application of R software. A total of 958 articles were identified and 26 studies demonstrating the effectiveness of the *Citrus* genus in controlling dyslipidemia were selected, of which 25 were included in the meta-analysis. The effects of *Citrus* products on dyslipidemia appear consistently robust, acting to reduce total cholesterol, LDL, and triglycerides, in addition to increasing HDL. These effects are associated with the composition of the extracts, extremely rich in antioxidant, as flavonoids, and that act on biochemical targets involved in lipogenesis and beta-oxidation. The risk of bias over all of the included studies was considered critically low to moderate. The meta-analysis demonstrated results favorable to control dyslipidemia by *Citrus* products. On the other hand, high heterogeneity values were identified, weakening the evidence presented. From this study, one can suggest that *Citrus* species extracts are potential candidates for dyslipidemia control, but more studies are needed to increase the strength of this occurrence.

**Systematic Review Registration**: [https://www.crd.york.ac.uk/prospero/display_record.php?ID=CRD42019121238], identifier [PROSPERO 2019 CRD42019121238].

## Introduction

Dyslipidemia has high rates of occurrence in the world population (Pirillo et al., 2021), being closely related to obesity, metabolic syndrome (Mach et al., 2020), atherosclerosis (Wiggins et al., 2019), coronary heart disease (Zhao et al., 2021), increased susceptibility to cancer (Khan et al., 2021), and more recently increased mortality and severity of COVID-19 (Atmosudigdo et al., 2021). This disorder is characterized by changes in the lipid profile, including an increase in total serum cholesterol, low-density lipoprotein (LDL-c), and triglycerides, as well as a decrease in high-density lipoprotein (HDL-c) rates in the blood (Fruchart et al., 2008). The relationships between these markers have been used as indicators of insulin resistance and metabolic disorders (Sowndarya et al., 2021), in addition to atherosclerosis and coronary heart disease (Abid et al., 2021). However, inflammation markers such as us-CRP (high serum sensitivity C-reactive protein) can also be considered important indicators to estimate the severity and risk of coronary artery disease (Patil et al., 2020). Although there are therapeutic options for the treatment of dyslipidemias, these are not fully effective, due to non-adherence to treatment by various factors such as adverse effects, intolerance, regimen complexity, and imperceptible benefits, besides the need to combine drugs to improve the clinical condition (Schulz, 2006; Ingersgaard et al., 2020). On the other hand, lipid-lowering drugs are still inaccessible to the majority of the population in low-income countries (Pirillo et al., 2021), making the search for new strategies to control dyslipidemia necessary.

In this sense, searching for new treatment strategies for this important health problem is necessary. In this perspective, several plants and natural products have been studied regarding their effects on dyslipidemia control (Ballard et al., 2019; Adel Mehraban et al., 2021); among them, the species of the genus *Citrus* (Lamiquiz-Moneo et al., 2020) stand out. Belonging to the Rutaceae family, the genus *Citrus* is widely distributed in tropical and subtropical regions (Manuel et al., 2020) and contains several substances with biological and nutritional potential, such as fibers (e.g., pectin), vitamins, and bioactive compounds, with emphasis on the flavonoids (Alam et al., 2013; Rafiq et al., 2018). Naringin, naringenin, nobiletin, narirutin, and hesperidin correspond to the most frequently found flavonoids. They have pronounced antioxidant and anti-inflammatory activities (Tripoli et al., 2007; Craft et al., 2012), in addition to being effective in controlling metabolic syndromes, lipid changes, and obesity (Geleijnse et al., 1999; Lee et al., 2001; Gattuso et al., 2007; Alam et al., 2013; Sahebkar, 2017; Ballard et al., 2019).

Thus, this review sought to compile the scientific findings that demonstrate the effect of *Citrus* extracts on the control of serum lipid levels, measuring the size of the effect through meta-analysis.

## Material and Methods

### Focused Question

The question to be answered was established from the bibliographic survey “Are species of the genus *Citrus* effective in reducing dyslipidemia?” conducted through four steps: (Pirillo et al., 2021) identification of the use of the *Citrus* species, (Mach et al., 2020) identification of the pathology to be applied (dyslipidemia), (Wiggins et al., 2019) definition of the types of studies included (preclinical and clinical), and (Zhao et al., 2021) definition of the target outcome to be analyzed, which is the lipid profile, building the PICOS strategy (patient or pathology, intervention, control, other outcomes, and the type of study). PICOS is highlighted as follows: P: dyslipidemia; I: species of the genera *Citrus* (extract); C: untreated or placebo-treated and hyperlipidemia-induced group; O: blood lipid levels; and S: preclinical or clinical studies.

### Review Writing and Registration of Protocols

The writing of this systematic review was based on the recommendations of the Preferred Reporting Items for Systematic Reviews and Meta-Analyses (PRISMA) (Page et al., 2021) tool. In addition, the instrument that guides how the experimental studies should be analyzed was ARRIVE (Animal Research: Reporting of *In Vivo* Experiments) guidelines (Kilkenny et al., 2010). The protocol for this review was registered in the International Prospective Register of Systematic Reviews (Prospero) database and registered on the website https://www.crd.york.ac.uk/prospero/, through approved registry No. CRD42019121238.

### Literature Search

The search was carried out through search strategies in the LILACS, PubMed, SciELO, and Scopus databases in 2019 and updated in March 2021. The terms used to compose the search in the databases were defined from consultations with MeSH and DeCS descriptors. Thus, the following search strategy was structured: “*CITRUS*” AND “Lipoproteins” OR “Cholesterol” OR “Epicholesterol” OR “Dyslipidemias” OR “Dyslipoproteinemia” OR “Hypercholesterolemia” OR “High Cholesterol Levels” OR “Hyperlipidemias” OR “Lipidemia,” described in detail in Supplementary Table S1.

### Study Selection and Eligibility Criteria

After excluding duplicate records, titles, abstracts, and full texts were independently analyzed by two researchers in order to determine the study’s eligibility for inclusion in the review. The inclusion criteria were preclinical studies or randomized clinical trials that include the use of *Citrus* species to assess the effect on the lipid profile. In this review, were excluded reviews, case studies, case reports, and studies that did not assess the action on the lipid profile, which included the use of juices from *Citrus* species and their action on the lipid profile, or the association of *Citrus* species with another compound that could modify the lipid profile, as well as studies that used compounds isolated from *Citrus* species to target hyperlipidemia. To assess the agreement among researchers, the statistical test of the Kappa coefficient (K) was applied.

### Data Extraction and Risk of Bias Assessment

Two independent reviewers extracted data from the included studies. The data from preclinical studies were as follows: *Citrus* species, type of extract and part of the plant, composition, hyperlipidemia induction model, evaluation methods, treatment, animal species, and results (all results that were in mg/dL were converted to mmol/L using the OnlineConversion.com electronic calculator according to the type of cholesterol). The data from clinical studies were as follows: *Citrus* species, type of extract and part of the plant, composition, study design/location, sample, criteria for inclusion and exclusion of participants, pathologies, treatment, and results (all results that were in mmol/L were converted to mg/dL using the OnlineConversion.com electronic calculator according to the type of cholesterol). All the outcomes of the experiments carried out in the articles were extracted for descriptive and inferential analyses.

Through ARRIVE, we apply the following: precise and concise description of the content of the article in the title, abstract, explanation of the methodological approach of the introduction, general and specific objectives, ethical nature of care and use of animals, study design regarding the number of animals per group, experimental procedures, information about animals such as sex, size, weight, and age, housing and breeding, sample size, statistical methods, description of results and their interpretation, and study funding.

All clinical studies included in this research were approved for methodological quality in the risk checklist of Cochrane randomized for controlled trials (Cochrane Training, 2019). Items such as generation of random sequence, concealment of allocation, certification of participants and professionals, as well as of evaluators, incomplete and selective outcomes, or whether the study presents any other problem or fraud were used. The studies considered as having the highest methodological quality were those related to randomization, blinding, and complete outcomes.

### Meta-Analysis

The studies selected for the meta-analysis had the following outcomes analyzed: total cholesterol, LDL, HDL, and triglyceride levels, including the baseline and post-treatment data from both the control and treatment groups for both preclinical and clinical studies. In addition to the primary outcomes, to improve the understanding of the effects observed in preclinical studies, the studies were separated into the following subgroups: route of administration of the extract, type of animal, type of extract, and parts of the plant used.

For the quantitative analysis of the articles, the studies selected presented the value of the sample n, mean, deviation, or standard error for the serum levels of total cholesterol, LDL, HDL, and/or triglycerides of the treatment and control groups. All data were tabulated in Excel and later analyzed using the application of R software. The heterogeneity of the studies was measured using Cochran’s Q test, using the I^2^ statistic, which was considered as heterogeneous when the *p* value was less than 0.05. The heterogeneity between the studies was defined using the I^2^ statistic, which was considered with an unimportant (I^2^ < 25%), moderate (25% < I^2^ < 75%), or high degree of heterogeneity (I^2^ > 75%) (Higgins and Thompson, 2002). For heterogeneous studies (I^2^ > 75%), the following subgroup analyses were performed: route of administration, type of animal, parts of the plant used in the extract, type of fruit, and type of extract. In addition, we performed a sensitivity analysis, sequentially removing the individual studies to determine whether any single study affected the overall effect estimate.

## Results

### Study Selection and Study Characteristics

During the search process, 958 articles were obtained: 169 from PubMed, 762 from SciVerse Scopus, 12 from SciELO, and 15 from LILACS. After analyzing the titles, 598 duplicate articles were removed. After excluding the repeated articles, 360 titles were screened for analysis according to the inclusion criteria, from which 329 studies were excluded for not inducing hyperlipidemia in an animal model or for not having dyslipidemia installed in the case of clinical studies. In addition, studies with isolated compounds of the *Citrus* species or without evaluation of total cholesterol, LDL-C, HDL-C, or triglycerides were also excluded.

**FIGURE 1:**
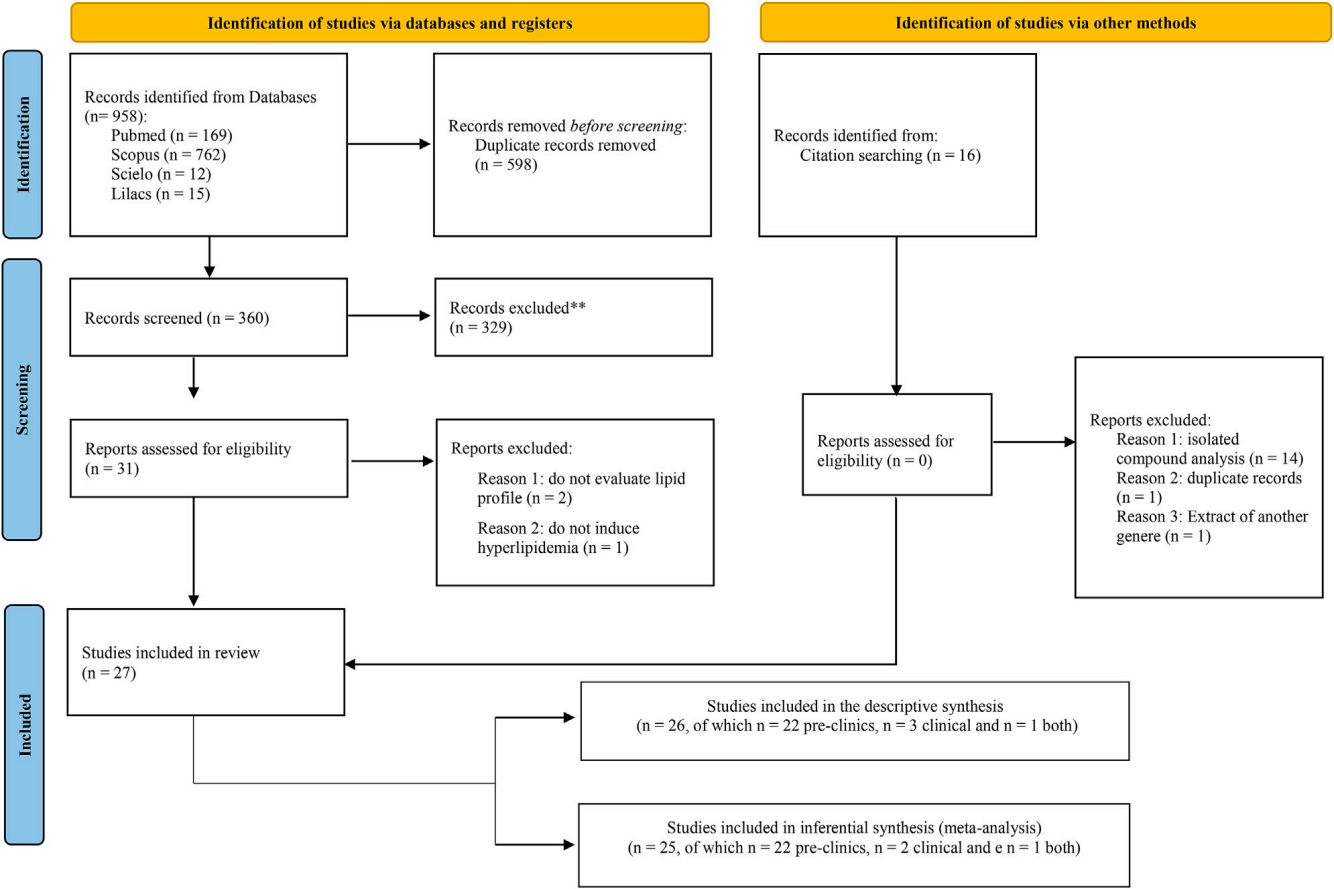
Flowchart of the studies included in the qualitative and quantitative synthesis.

After this design, 31 articles remained, the full texts of which were analyzed, thus yielding 27 articles that were finally included in the qualitative synthesis (Figure 1; Tables 1–3). Of these, 22 studies were preclinical trials (Vinson et al., 1998; Bok et al., 1999; Terpstra et al., 2002; Zulkhairi et al., 2010; Ding et al., 2012; Kang et al., 2012; Raasmaja et al., 2013; Lu et al., 2013; Kim et al., 2013; Muhtadi et al., 2015; Dinesh and Hegde, 2016; Shin et al., 2016; Ashraf et al., 2017; Fayek et al., 2017; Chou et al., 2018; Feksa et al., 2018; Mir et al., 2019; Sato et al., 2019; Hase-Tamaru et al., 2019; Ling et al., 2020; Ke et al., 2020; Lee et al., 2020), 3 were exclusively clinical studies (Gorinstein et al., 2007; Toth et al., 2015; Cai et al., 2017) and 1 study contained preclinical and clinical protocols (Mollace et al., 2011) (Figure 1). For the quantitative synthesis, 25 articles (Vinson et al., 1998; Bok et al., 1999; Gorinstein et al., 2007; Zulkhairi et al., 2010; Mollace et al., 2011; Ding et al., 2012; Kang et al., 2012; Terpstra et al., 2012; Kim et al., 2013; Lu et al., 2013; Raasmaja et al., 2013; Muhtadi et al., 2015; Dinesh and Hegde, 2016; Shin et al., 2016; Ashraf et al., 2017; Cai et al., 2017; Fayek et al., 2017; Chou et al., 2018; Feksa et al., 2018; Hase-Tamaru et al., 2019; Mir et al., 2019; Sato et al., 2019; Ke et al., 2020; Lee et al., 2020; Ling et al., 2020) were selected. The level of agreement among the reviewers was 0.470, being considered as moderate.

**TABLE 1:** Detailed description of the preclinical studies of the effect of *Citrus* extract on hyperlipidemia included in the systematic review.

References, country	Extract, plant part, and species	Composition	Model	Evaluated parameters	Treatment protocol	Animal (n/group)
Vinson et al.,	Hydroalcoholic extract of whole dried ripe fruits *C. aurantium*	25.7% ascorbic acid	Hamster fed on a high-cholesterol diet	LDL, VLDL	Feed containing 3% of the extract or 4% of the extract associated with ascorbic acid (57 mmol/kg diet) daily, for 4 or 10 weeks	Male
1998 (Vinson et al., 1998)		9.9% flavonoids (quercetin, hesperidin, naringenin, and myricetin)		HDL, TC, TG, foam cell injury		Golden Syrian
EUA		31.2% protein		in the aorta artery		Hamsters (n = 10)
		3.2% ash		lipid peroxidation		
		30% carbohydrates				
Bok et al.,	Hydroalcoholic extract of the peel *C. reticulata*	2.7 g of protein	Rats fed on a high-cholesterol diet	Plasmatic and hepatic TC, TG, HDL, LDL	16.7 g/100 g of diet for 6 weeks	Male
1999 (Bok et al., 1999)		1.8 g of fat		AI^a^, fecal neutral sterols, HMGR, and ACAT activities in liver tissue		Sprague Dawley rats (n = 10)
Korea		1.0 g of ash				
		20 g of fructose				
		16.5 g of glucose				
		8.6 g of sucrose				
		0.6 g of hesperidin				
		0.03 g of naringin and 9.67 g of other sugars				
Terpstra et al., 2002 (Terpstra et al., 2002)	Peels or waste stream material of *C. limon*	-	Hamster fed on a high-cholesterol diet	BW, FI, and liver weight	Diets containing 3% of cellulose or lemon peels or the waste stream of the lemon pectin extraction	Male hybrid
Netherlands				TC of plasma and liver	for 8 weeks	F_1_B Golden
				Plasmatic TG, LDL, HDL, VLDL		Syrian
				bile acids, and fecal sterols		Hamster (n = 14)
Mollace et al., 2011 (Mollace et al., 2011)	Polyphenolic fraction of *C. bergamia* Risso & Poiteau peeled-off fruits	Neoeriocitrin (77,700 ppm), naringin (63,011 ppm), neohesperidin (72,056 ppm), melitidine (15,606 ppm), and brutieridine (33,202 ppm)	High-cholesterol diet-induced hyperlipemia	BW, TC, LDL, HDL	10 or 20 mg/kg daily (p.o.)	Male
Italy				TG and glucose	for 30 days	Wistar
				Neutral sterols and fecal bile acids		Rats (n = 10)
Zulkhairi et al., (Zulkhairi et al., 2010)	Aqueous extract (5% and 10%) of dried whole fruits *C. mitis*	Phenolic compounds	Rats fed on a high-cholesterol diet	BW, TC, HDL, LDL, TG, AI^b^, sdLDL^c^	5 mg/kg of extract at 5% and 10%	Male
Malaysia				Scavenging activity of DPPH radicals, reducing power, lipid peroxidation (*in vitro*)	daily (p.o.)	Sprague Dawley rats (n = 6)
					for 10 weeks	
Ding et al.,	Hydroalcoholic extract of *C. ichangensis* peel	Naringin, hesperidin, poncirin, neoeriocitrin	High-fat diet-induced	BWG, FI	Diet supplemented with 1% of extract, for 8 weeks	Female
2012 (Ding et al., 2012)		narirutin, neohesperidin, naringenin, nobiletin,	Obese	TC, TG, LDL, HDL, and glucose		C57BL/6 mice (n = 7)
China		and tangeretin		Fecal and hepatic TC and TG; size of EWAT; mRNA expression of PPARγ, LXR, and them target genes in liver tissue		
Kang et al.,	Hydroalcoholic extract of *C*	Tangeretin (55.13 mg/g)	High-fat diet-induced	BWG, FI	150 mg/kg/day of extract (p.o.)	Male
2012 (Kang et al., 2012)	*sunki* peel	Nobiletin (38.83 mg/g)	Obese	TC, TG, GPT, GOT, and LDH, EPAT weight, liver fat; p-AMPK, p-ACC, and adiponectin mRNA expression in EAT.	for 70 days	C57BL/6 mice (n = 10)
Korea		Hesperidin (17.11 mg/g)		In mature 3T3-L1 adipocytes: LKB1, AMPK, ACC, PKA, and HSL phosphorylation, CPT-1a gene expression, and glycerol release		
		Rutin (17.02 mg/g)				
		Sinensetin (4.23 mg/g)				
Raasmaja et al., 2013 (Raasmaja et al., 2013)	Hydroalcoholic extract of *C. grandis* (L.) Osbeck whole fruits	Naringin at 19%	High-fat diet-induced	BWG, FI	300, 600, or 1,200 mg/kg (p.o.) daily	Female
Finland			Obese	TG, TC, HDL, glucose, insulin, ghrelin, GLP-1	for 12 weeks	Zucker
				PYY, leptin, and amylin in plasma		Rats (n = 10)
Lu et al.,	Hydroalcoholic extract of *Citrange* (*Poncirus trifoliata* x *C. sinensis*) peel or flesh and seed	Bark extract	High-fat diet-induced obese	BWG, FI, ipGTT, blood glucose, serum TG, TC, LDL and HDL, hepatic TG and TC	Diet supplemented with 1% w/w of peel extract	Female
2013 (Lu et al., 2013)		Neoeriocitrin (14.5 mg/g), naringin (8.12 mg/g), neohesperidin (21.1 mg/g), and poncirin (14.1 mg/g)		Fecal TC and TG, histological analysis	or 1% w/w of flesh and seed	C57BL/6 mice (n = 6)
China		Seed extract		of liver tissue	extract, daily	
		Poncirin (4.85 mg/g) Neohesperidin (1.87 mg/g) Naringin (0.87 mg/g)		mRNA levels of PPARγ, LXR, and their target genes in liver tissue	for 8 weeks	
Kim et al.,	Hydroalcoholic extract of *C. junos* Tanaka peel	Hesperidin (36.3 mg/100 g)	High-fat diet-induced obese	BWG, FI	Diet supplemented with 1% and 5% of extract	Male
2013 (Kim et al., 2013)		Naringin (11.6 mg/100 g)		TC, TG, glucose, insulin, leptin, resistin, GOT, GPT, histological analysis of liver tissue	for 9 weeks	C57BL/6 J mice (n = 8)
Korea		Rutin (2.7 mg/100 g)		AMPK phosphorylation in muscle tissue		
		Quercetin (1.7 mg/100 g) and tangeretin (0.7 mg/100 g)		AMPK and PPARγ activation in C2C12 and HEK293 cells, respectively		
Muhtadi et al., 2015 (Muhtadi et al., 2015)	Hydroalcoholic extract of *C. sinensis* fruit peel	-	High-fat diet-induced hypercholesterolemia	TC; glucose in rats	125, 250, and 500 mg/kg (p.o.), daily for 2 weeks	Male
Indonesia				induced by alloxan monohydrate	After 4-week diet	Wistar rats (n = 5)
Dinesh and Hegde, 2016 (Dinesh and Hegde, 2016)	Hydroalcoholic extract of *C. maxim*a leaves	Flavonoids, alkaloids, carbohydrates, glycosides, saponins, and tannins	Cafeteria diet and Olanzapine-induced obesity	BWG, FI	200 and 400 mg/kg (p.o.), daily for 4 weeks	Female
India				TC, TG, HDL, LDL, VLDL, GOT, GPT, glucose		Wistar rats (n = 6)
				Liver weight and TG		
Shin et al.,	Hydroalcoholic extract of *C. junos* Tanaka peel	-	Mice fed on a high-cholesterol diet	BWG, FI	Diet supplemented with 1% and 5% of the extract	Male
2016 (Shin et al., 2016)				TG, TC, HDL, GOT, GPT, ALP, histological analysis	for 10 weeks	C57BL/6 J mice (n = 8)
Korea				of liver tissue		
				Expression of PPARα, FAS, and HMGR in liver tissue		
				Lipid accumulation and expression of p-AMPK, p-ACC, PPARα, CPT-1, and HMGR in HepG2 cells		
Ashraf et al.,	Hydroalcoholic extract of *C. sinensis* peel	-	Rats fed on high-glucose or cholesterol-rich diet	BWG, FI	Diet supplemented with 10% *Citrus* peel powder (functional) and 5% peel extract (nutraceutical), for 8 weeks	Male
2017 (Ashraf et al., 2017)				TG, TC, LDL, HDL, glucose, insulin		Sprague
Pakistan						Dawley rats (n = 6)
Fayek et al.,	Methanolic extract, hexanic extract, aqueous homogenate of *C. reticulata* (Mandarin), *C. sinensis* (sweet orange), *C. paradise* (white grapefruit), or *C. aurantiifolia* (lime) fruit peels	Nobiletin (%) in hexanic extracts	Hypercholesterolemia induced by diet rich in cholesterol and bile salts	TC	0.1 ml of the corresponding extract (p.o.) for 8 weeks	Male
2017 (Fayek et al., 2017)		Mandarin (10.14%)		TG and glucose		Wistar rats (n = 6)
Egypt		Sweet orange (3.6%)				
		White grapefruit (0.9%)				
		Lime (0.0045%)				
		Pectin (%) in peel powder				
		Sweet orange (21.33%)				
		Lime (19.7%)				
		While grapefruit (11.66%)				
		Mandarin (9.14%)				
Chou et al., 2018 (Chou et al., 2018)	Methanolic extract of *C. reticulata*	Narirutin (4.52 ± 0.31 mg/g), hesperidin (9.14 ± 0.32 mg/g), nobiletin (2.54 ± 0.07 mg/g)	High-fat diet-induced	AST, ALT, triglyceride, total cholesterol, glucose, insulin, HOMA-IR	1% of the corresponding extract for 11 weeks	Male
China		Tangeretin (1.67 ± 0.05 mg/g)	obese			C57BL/6 J mice (n = 8)
Feksa et al., 2018 (Feksa et al., 2018)	Hydroalcoholic extract of leaves of *C. maxima*	Gallic acid, catechin, caffeic acid, epicatechin, rutin and isoquercetin, and the major compounds	High-fat diet and fructose	Blood count, AST, ALT, triglyceride, total cholesterol, LDL, HDL, glucose, urea, creatinine,	50 mg/kg	Male
Brazil		were caffeic acid (3.71 mg/g) and catechin (3.65 mg/g				Wistar rats (n =
Mir et al., 2019 (Mir et al., 2019)	Hydroalcoholic extract of *C. latifolia*	-	Hypercholesterolemia induced by diet rich in cholesterol	triglyceride, and total cholesterol	1% of the corresponding extract for 4 weeks	Male
Algeria						Wistar rats (n = 10)
Sato et al., 2019 (Sato et al., 2019)	*C. tumida* peel powder	Calorie (275 kcal), moisture (2.9 g), protein (7.4 g), fat (2.7 g), ash (4.9 g), carbohydrate (82.1 g), sugar (28.4 g), fiber (53.7 g), galacturonic acid (12.2 g), and sodium (4.3 mg)	High-fat diet	AST, ALT, triglyceride, total cholesterol, HDL-C, creatinine, albumin, calcium, and LDH	*C. tumida* peel powder 5% (w/w)	Male
Japan						C57BL/6 J mice (n = 8)
Tamaru et al., 2019 (Hase-Tamaru et al., 2019)	*C. unshiu* MARC lyophilized and powdered	76.1 g carbohydrate, 7.6 g crude protein, 0.7 g crude fat, 2.7 g ash, 12.9 g moisture, 40.9 g	High-fat diet	Total cholesterol, triglycerides, free fatty acids, glucose, insulin, and leptin	2.5%	Sprague Dawley (SD) rats (n = 7)
Japan		total fiber, 6.6 g total pectin, 14.4 g hesperidin, and 3.0 g narirutin			5.0%, or 10.0%	
Lee et al., 2020 (Lee et al., 2020)	*C. unshiu*: dried extract (CPEW) and lyophilized (CPEF)	Hesperidin, narirutin, and synephrine	High-fat diet	AST, ALT, triglyceride, total cholesterol, and LDL-C	CPEW: 50 mg/kg; 100 mg/kg	Male
Korea					CPEF: 50 mg/kg; 100 mg/kg	SD rats (n = 8)
Ling et al., 2020 (Ling et al., 2020)	*C. changshan-huyou*	Naringin, narirutin, and neohesperidin	High-fat diet	AST, ALT, triglyceride, total cholesterol, LDL-C, and HDL-C	PTFC: 25 mg/kg; 50 mg/kg; 100 mg/kg	Golden hamsters (n = 12)
China						
Ke et al., 2020 (Ke et al., 2020)	*C. reticulata* Blanco	Nobiletin (98.34 mg/g), heptamethoxyflavone (44.26 mg/g), tangeretin (26.20 mg/g), and isosinensetin (26.14 mg/g)	High-fat diet	Triglyceride, total cholesterol, LDL-C, and HDL-C	0.2 and 0.5% JZE	C57BL/6 J mice (n = 8)
China						

**glutamic:** p.o., intragastric gavage; TC, total cholesterol; TG, triglycerides; LDL, low-density lipoprotein; HDL, high-density lipoprotein; VLDL, very low-density lipoprotein; LDH, lactate dehydrogenase; GOT, -oxaloacetic transaminase; GPT, glutamic-pyruvic transaminase; EWAT, epididymal white adipose tissue; PPARγ, peroxisome proliferator-activated receptor γ; FAS, fatty acid synthase; ACO, acyl-CoA oxidase; LXRα, liver X receptor α; LXRβ, liver X receptor β; AMPK, AMP-activated protein kinase; ACC, acetyl-CoA carboxylase; PKA, cAMP-dependent protein kinase; HSL, hormone-sensitive lipase. GLP-1, glucagon-like peptide-1; PYY, pancreatic peptide YY; BWG, body weight gain; FI, food intake; ipGTT, intraperitoneal glucose tolerance test; ALP, alkaline phosphatase; FAS, fatty acid synthase receptor; CPT-1, carnitine palmitoyl transferase-1; HMGR, 3-hydroxy-3-methylglutaryl-coenzyme A reductase; EPAT, epididymal and perirenal adipose tissue; EAT, epididymal adipose tissue.

a The duration of the experiment is not explicitly informed in the article. AI, atherogenic index.

b [(TC-HDL)/HDL].

c (LDL/HDL); sdLDL, small dense LDL, particle size.

d (TG/HDL).

**TABLE 2:** Outcomes of the preclinical studies included in this systematic review.

Reference	Experimental group (mmol/L)	Control group (mmol/L)	Summary of results
Vinson et al., (Vinson et al., 1998)	Baseline: TC: 5.84; HDL: 3.31; TG: 25.1	Baseline: TC: 10.3; HDL: 2.84; TG: 41.6	↓TC and TG
	10 weeks: TC: 6.88; HDL: 1.68; TG: 27.1	10 weeks: TC: 15.1; HDL: 1.48; TG: 55.9	↓ lipid peroxidation
			↓ atherosclerosis signals (↓ area and density of foam cells), without changing BW
Bok et al. (Bok et al., 1999)	Baseline	Baseline:	↓ plasma TC
	6 weeks: TC: 2.44; HDL: 0.61; TG: 1.22	6 weeks: TC: 3.8; HDL: 0.57; TG: 1.12	↓ hepatic TC and TG, without changing HDL, TG, and LDL plasmatic
			↓AI and cholesterol excretion
			↓ HMGR and ACAT activities
Terpstra et al. (Terpstra et al., 2002)	Baseline:	Baseline:	↓ plasma and liver TC, ↓ VLDL + LDL being more effective in ↓VLDL, without changing HDL, ↑ excretion of fecal neutral sterols and bile acids
	8 weeks (lemon peel): TC: 3.51	8 weeks (cellulose): TC: 4.21	without changing BW, FI, and liver weight
	8 weeks (waste stream): TC: 3.44		
Mollace et al. (Mollace et al., 2011)	Baseline:	Baseline:	↓TC, LDL, and TG, without changing BW, HDL and glucose
	30 days (10 mg): TC: 5.95; LDL: 4.49; HDL: 0.58; TG: 2.75	30 days: TC: 8.19; LDL: 6.04; HDL: 0.53; TG: 2.74	↑ fecal neutral sterols and bile acids
	30 days (20 mg): TC: 5.00; LDL: 3.90; HDL: 0.65; TG: 2.74		
Zulkhairi et al. (Zulkhairi et al., 2010)	Baseline (5%)	Baseline: TC: 1.75; LDL: 0.45; HDL: 0.85; TG: 0.54	↓ TC, LDL, TG
	TC: 1.73; LDL: 0.45; HDL: 1.34; TG: 0.76	4 weeks	↑ HDL
	Baseline (10%)	TC: 2.13; LDL: 0.93; HDL: 0.89; TG: 0.79	↓AI and sdLDL
	TC: 1.68; LDL: 0.49; HDL: 1.27; TG: 0.74		Antioxidant activity, without changing BW
	4 weeks (5%)		
	TC: 1.28; LDL: 0.27; HDL: 1.39; TG: 0.63		
	4 weeks (10%)		
	TC: 1.06; LDL: 0.23; HDL: 1.54; TG: 0.53		
Ding et al. (Ding et al., 2012)	Baseline:	Baseline:	↓ BWG
	8 weeks	8 weeks	↓TC and LDL plasmatic
	TC: 2.27; LDL: 0.35; HDL: 2.32; TG: 0.70	TC: 2.65; LDL: 0.46; HDL: 1.95; TG: 0.70	↓ hepatic TC, TG, glucose, and adipocyte size, without changing
			Plasmatic FI, HDL, and TG and
			fecal TC and TG
			↓ expression of PPARγ (↓FAS, ACO, and UCP2 and ↑ CD36) ↓ LXR α and β (↓ ApoE, CYP7A1, LPL, and ↑ABCA1)
Kang et al. (Kang et al., 2012)	Baseline:	Baseline:	↓ BWG without changing in FI
	70 days	70 days: TC: 4.63; TG: 1.56	↓ TC, TG, LDH, GOT, and GPT
	TC: 3.81; TG: 0.94		↓ weight and cell size of EPAT
			↓ liver fat
			↑ p-AMPK, p-ACC, p-LKB1, and adiponectin
			↑ glycerol release
			↑ p-PKA and p-HSL
Raasmaja et al. (Raasmaja et al., 2013)	Baseline (300 mg/kg)	Baseline	Tendency to ↓ TC, glucose, and TG and ↑ HDL
	TC: 3.72; HDL: 1.42; TG: 8.34	TC: 3.56; HDL: 1.67; TG: 7.31	↓ GLP-1 and reversing the ↓ of ghrelin, without changing BWG, FI
	Baseline (600 mg/kg)	12 weeks	PYY, leptin, insulin, and amylin
	TC: 3.13; HDL: 1.70; TG: 6.27	TC: 4.13; HDL: 0.52; TG: 15.76	
	Baseline (1,200 mg/kg)		
	TC: 3.59; HDL: 1.53; TG: 8.11		
	12 weeks (300 mg/kg)		
	TC: 4.23; HDL: 0.44; TG: 16.68		
	12 weeks (600 mg/kg)		
	TC: 3.62; HDL: 0.80; TG: 12.57		
	12 weeks (1,200 mg/kg)		
	TC: 4.36; HDL: 0.80; TG: 17.42		
Lu et al. (Lu et al., 2013)	Baseline	Baseline	↓ BWG
	8 weeks (peel)	8 weeks	Improves glucose tolerance and insulin resistance
	TC: 2.30; LDL: 0.36; HDL: 2.00; TG: 0.70	TC: 2.64; LDL: 0.41; HDL: 1.97; TG: 0.70	↓ serum glucose, TC, and LDL
	8 weeks (seed)		↓ hepatic TC and TG, without changing FI, serum HDL, and fecal TC and TG
	TC: 2.43; LDL: 0.41; HDL: 1.87; TG: 0.74		↓ PPARγ (↓ ap2, FAS); ↓ LXRβ (↓ LPL and ApoE and ↑ ABCG1)
			↓ lipid accumulation in liver tissue
Kim et al. (Kim et al., 2013)	Baseline	Baseline:	↓ BWG, glucose, TG, TC, insulin, leptin, and resistin
	9 weeks (1%)	9 weeks	↑ glucose uptake
	TC: 2.00; TG: 0.85	TC: 2.37; TG: 0.88	↓ liver tissue fat
	9 weeks (5%)		↑ PPARγ and AMPK, without changing FI, GOT, and GPT
	TC: 1.91; TG: 0.76		
Muhtadi et al. (Muhtadi et al., 2015)	Baseline (125 mg/kg): TC: 4.31	Baseline: TC: 3.77	↓ TC and glucose
	Baseline (250 mg/kg): TC: 5.08	2 weeks: TC: 3.27	
	Baseline 500 (mg/kg): TC: 4.87		
	2 weeks (125 mg/kg): TC: 1.88		
	2 weeks (250 mg/kg): TC: 2.13		
	2 weeks (500 mg/kg): TC: 2.02		
Dinesh and Hegde (Dinesh and Hegde, 2016)	Baseline	Baseline:	↓ BWG and FI
	4 weeks (200 mg/kg)	4 weeks	↓ TC, TG, LDL, and VLDL
	TC: 79.76; LDL: 54.31; HDL: 40.68; TG: 104.3	TC: 88.75; LDL: 74.71; HDL: 35.11; TG: 130.0	↑ HDL
	4 weeks (400 mg/kg)		↓ GOT and GPT
	TC: 75.77; LDL: 51.75; HDL: 43.22; TG: 98.05		↓ liver weight and TG
			↓ glucose
Shin et al. (Shin et al., 2016)	Baseline:	Baseline:	↓ BWG
	10 weeks (1%)	10 weeks	↓ TC, LDL, GOT, GPT, ALP, without changing FI, HDL
	TC: 2.89; LDL: 1.81; HDL: 0.87	TC: 4.03; LDL: 3.03; HDL: 0.80	↓ liver fat content and weight
	10 weeks (5%)		↑ p-AMPK, p-ACC, PPARα, and CPT-1 expression
	TC: 2.96; LDL: 1.80; HDL: 0.80		↓ FAS and HMGR expression
			↓ lipid accumulation
Ashraf et al. (Ashraf et al., 2017)	Baseline (powder)	Baseline	Tendency to
	TC: 3.34; HDL: 1.19; LDL: 1.67; TRI: 1.07	TC: 3.30; HDL: 1.17; LDL: 1.63; TRI: 1.04	↓ BWG and FI
	Baseline (extract)	8 weeks	↓ TG, TC, and LDL
	TC: 3.32; HDL: 1.21; LDL: 1.62; TRI: 1.05	TC: 3.81; HDL: 1.17; LDL: 1.85; TRI: 1.16	↑ HDL
	8 weeks (powder)		↓ glucose and ↑ insulin
	TC: 3.14; HDL: 1.21; LDL: 1.52; TRI: 1.01		
	8 weeks (extract)		
	TC: 3.03; HDL: 1.24; LDL: 1.44; TRI: 0.97		
Fayek et al. (Fayek et al., 2017)	Baseline:	Baseline:	Tendency to ↓ TC
	Tangerine (alcoholic extract)	Diet	↓ TG and glucose
	TC: 2.00; TG: 0.78	TC: 3.92; TG: 2.66	
	Orange (alcoholic extract)		
	TC: 3.25; TG: 0.94		
	Hybrid (alcoholic extract)		
	TC: 3.95; TG: 0.85		
	Lime (alcoholic extract)		
	TC: 5.47; TG: 0.51		
Chou et al. (Chou et al., 2018)	Baseline:	Baseline:	Tendency to ↓ TC
	11 weeks (1%)	11 weeks (diet)	↓ TG and insulin resistance
	TC: 3.85; TG: 0.44	TC: 4.68; TG: 0.85	
Feksa et al. (Feksa et al., 2018)	Baseline	Baseline:	Tendency to
	45 days (50 mg/kg)	45 days (diet): TC: 3.34; TG: 3.38; HDL: 0.47; LDL: 1.23	↓ TG, TC, and LDL
	TC: 2.12; TG: 2.84; HDL: 0.34; LDL: 0.61		
Mir et al. (Mir et al., 2019)	Baseline	Baseline:	Tendency to
	4 weeks (1%)	4 weeks (diet)	↓ TG and TC
	TC: 3.8; TG: 0.9	TC: 5.9; TG: 1.8	
Sato et al. (Sato et al., 2019)	Baseline:	Baseline:	Tendency to
	4 weeks (5%)	4 weeks (diet)	↓ TG and TC
	TC: 3.31; TG: 0.28; HDL: 2.06	TC: 4.39; TG: 0.41; HDL: 2.42	
Tamaru et al. (Hase-Tamaru et al., 2019)	Baseline:	Baseline:	Tendency to
	4 weeks (2.5%)	4 weeks (diet)	↓ TG and TC
	TC: 2.01; TG: 1.67	TC: 2.27 TG: 2.00	↓ free fatty acids, glucose, insulin, and leptin
	4 weeks (5%)		↓ FAS, G6PDH in cytosol, and PAP in microsome
	TC: 2.22; TG: 1.63		
	4 weeks (10%)		
	TC: 1.72; TG: 2.74		
Lee et al. (Lee et al., 2020)	Baseline	Baseline:	Tendency to
	8 weeks (CPEW 50 mg/kg): TC: 4.00; TG: 2.89; LDL: 2.58	8 weeks (diet): TC: 4.00; TG: 2.89; LDL: 2.58	↓ TG and TC
	8 weeks (CPEW 100 mg/kg): TC: 3.54; TG: 2.52; LDL: 2.27		
	8 weeks (CPEF 50 mg/kg): TC: 4.08; TG: 2.79; LDL: 2.56		
	8 weeks (CPEF 100 mg/kg): TC: 3.64; TG: 2.59; LDL: 2.37		
Ling et al. (Ling et al., 2020)	Baseline	Baseline:	Tendency to
	4 weeks (25 mg/kg): TC: 32.00; TG: 10.20; HDL: 2.30; LDL: 11.41	4 weeks (diet)	↓ TG, TC, and LDL-C
	4 weeks (50 mg/kg): TC: 22.30; TG: 5.30; HDL: 2.83; LDL: 9.83	TC: 41.59; TG: 11.15; HDL: 4.95; LDL: 11.80	
	4 weeks (100 mg/kg): TC: 21.70; TG: 5.30; HDL: 2.65; LDL: 8.67		
Ke et al. (Ke et al., 2020)	Baseline	Baseline:	Tendency to
	4 weeks (0.2%): TC: 5.69; TG: 0.28; HDL: 4.10; LDL: 1.01	4 weeks (diet)	↓ TG, TC, and LDL-C
	4 weeks (0.5%): TC: 5.04; TG: 0.28; HDL: 3.84; LDL: 0.81	TC: 5.62; TG: 0.41; HDL: 4.20; LDL: 1.20	

TC, total cholesterol; TG, triglycerides; LDL, low-density lipoprotein; HDL, high-density lipoprotein; VLDL, very low-density lipoprotein; BW, body weight; HMGR, 3-hydroxy-3-methylglutaryl-coenzyme A reductase; ACAT, acyl-CoA cholesterol acyltransferase; AI, atherogenic index; FI, food intake; BWG, body weight gain; PPARγ, peroxisome proliferator-activated receptor γ; FAS, fatty acid synthase; ACO, acyl-CoA oxidase; UCP2, uncoupling protein 2; CD36, cluster of differentiation 36; LXR, liver X receptor; ApoE, apolipoprotein E; CYP7A1, cholesterol 7α-hydroxylase; LPL, reducing lipoprotein lipase; ABCA1, ATP-binding cassette transporter A1; LDH, lactate dehydrogenase; GPT, glutamic-pyruvic transaminase; GOT, glutamic-oxaloacetic transaminase; AMPK, AMP-activated protein kinase; ACC, acetyl-CoA carboxylase; PKA; AMP-dependent protein kinase; HSL, hormone-sensitive lipase; PYY, pancreatic peptide YY; GLP-1, glucagon-like peptide-1; ABCG1, ATP-binding cassette transporter G1; ALP, alkaline phosphatase; CPT-1, carnitine palmitoyl transferase-1; G6PDH, glucose-6-phosphate dehydrogenase; PAP, phosphatidic acid phosphohydrolase in the microsome.

**TABLE 3:** Detailed description of the clinical studies of the effect of *Citrus* extract on hyperlipidemia included in the systematic review.

References/country	Extract, plant part and species	Composition	Sample	Pathology	Parameters evaluated	Treatment protocol
Gorinstein et al.,	Fresh fruit peels of red grapefruit or blond grapefruit processed	Anthocyanins	57 patients (39–72 years)	Hypertriglyceridemia and coronary	HR, BP, BW	Daily supplementation with red or blond grapefruits associated with anti-atherosclerosis diet for 30 days (n = 19/group)
2007 (Gorinstein et al., 2007)		Red: 51.5 mg/100 g		disease	CT, LDL, HDL,	
Israel		Blond: 49.3 mg/100 g			TG, serum antioxidant activity by ABTS and TEAC	
		Flavonoids (naringin)				
		Red: 21.61 mg/100 g				
		Blond: 19.53 mg/100 g				
		Total fibers				
		Red: 1.39 g/100 g				
		Blond: 1.37 g/100 g				
Mollace et al., 2011 (Mollace et al., 2011)	Polyphenolic fraction of *C. bergamia* peeled-off fruits	Neoeriocitrin (77,700 ppm)	237 patients	Hyperlipemia associated or not with hyperglycaemia	TC, LDL, HDL,	500 or 1,000 mg/day encapsulated with 50 mg ascorbic acid, for 30 days (n = 104–32/group)
Italy		Naringin (63,011 ppm)			TG, reactive vasodilation	
		Neohesperidin (72,056 ppm) and melitidine (15,606 ppm)				
		Brutieridine (33,202 ppm)				
Toth et al., 2016 (Toth et al., 2015)	Bergavit^®^ (Bergamot juice derived extract, *C. bergamia*)	150 mg of flavonoids	80 individuals (42 men and 38 women)	Moderate hypercholesterolemia	TC, LDL, HDL, TG, VLDL, IDL, IMT, LDL size	150 mg/day for 6 months (n = 80)
Italy		16% of neoeriocitrin				
		47% neohesperidin				
		37% naringin				
Cai et al., 2017 (Cai et al., 2017)	*C. bergamia* extract (CitriCholess^®^)	25% bioflavonoids, sterols and orange oil (820 mg/day), vitamin C (50 mg/day), vitamin B6 (20 mg/daily), B12 (2,000 µg/day), and folic acid (800 µg/day)	98 older people	Dyslipidemia and arterial hypertension and problems of glucose intolerance	TG, TC, LDL, HDL, glucose, BW, WC, HC, WHR, and BMI	500 mg/day for 12 weeks (n = 48–50/group)
China						

Legend: TC, total cholesterol; TG, triglycerides; LDL, low-density lipoprotein; HDL, high-density lipoprotein; TEAC, Trolox-equivalent antioxidant capacity; HR, heart rate; BP, blood pressure; BW, body weight; IMT, carotid intima-media thickness; BW, body weight (kg); WC, waist circumference (cm); HC, hip circumference (cm); WHR, waist-to-hip ratio; BMI, body mass index.

Tables 2 and 3 show the general characteristics and results of the preclinical studies, arranged in the chronological order of publication. Table 4 present the experimental conditions and results of clinical trials also arranged in the chronological order.

**TABLE 4:** Outcomes of the clinical studies included in this systematic review.

Reference	Experimental group (mg/dL)	Control group (mg/dL)	Summary of results
Gorinstein et al. (Gorinstein et al., 2007)	Baseline:	Baseline:	Red: ↓ TC, LDL, and TG
	Red	TC: 306.26	Blond: ↓ LDL only
	TC: 258.70	LDL: 243.23	Both: ↑ serum antioxidant activity, without change in HR, BP, BW,
	LDL: 193.73	HDL: 46.20	HDL
	HDL: 52.59	TG: 205.49	
	TG: 149.68		
	Blond		
	TC: 283.06		
	LDL: 217.32		
	HDL: 50.27		
	TG: 193.97		
Mollace et al. (Mollace et al., 2011)	Baseline (500 mg)	Treated with capsules containing	↓ TC, TG, and LDL
	TC: 286.00	500 mg of maltodextrin and 50 mg of ascorbic acid	↑ HDL
	LDL: 184.96	Baseline	↓ glucose
	HDL: 34.55	TC: 275.67	↑ reactive vasodilation
	TG: 266.87	LDL: 186.31	
	Baseline (1,000 mg)	HDL: 34.59	
	TC: 279.40	TG: 275.62	
	LDL: 189.70	TC: 279.40	
	HDL: 32.78	LDL: 185.64	
	TG: 270.11	HDL: 35.05	
	After 30 days (500 mg)	TG: 275.71	
	TC: 211.42		
	LDL: 132.79		
	HDL: 40.53		
	TG: 180.18		
	After 30 days (1,000 mg)		
	TC: 201.99		
	LDL: 125.34		
	HDL: 46.00		
	TG: 157.48		
Toth et al. (Toth et al., 2015)	Baseline	Baseline:	↓ TC, LDL, TG, and IMT
	TC: 224.28	TC: 255.22	↑ HDL, IDL, and LDL size
	LDL: 143.07	LDL: 177.88	without changing VLDL
	HDL: 54.13	HDL: 50.27	
	TG: 132.86	TG: 159.43	
Cai et al. (Cai et al., 2017)	Baseline	Baseline	↓ LDL
	TC: 211.13	TC: 217.32; LDL: 138.43; HDL: 51.81; TG: 170.94	↓ BW, WC,
	LDL: 131.09	TC: 210.36	WHR, and BMI
	HDL: 49.88	LDL: 132.63	without changing TG, TC, HDL, glucose, HC
	TG: 192.20	HDL: 52.20; TG: 172.71	
	500 mg		
	TC: 198.76		
	LDL: 121.03		
	HDL: 50.27		
	TG: 162.09		

Legend: TC, total cholesterol; TG, triglycerides; LDL, low-density lipoprotein; HDL, high-density lipoprotein; TEAC, Trolox-equivalent antioxidant capacity; HR, heart rate; BP, blood pressure; BW, body weight; IMT, carotid intima-media thickness; BW, body weight (kg); WC, waist circumference (cm); HC, hip circumference (cm); WHR, waist-to-hip ratio; BMI, body mass index.

The selected articles were published between 1998 and 2020, with a predominance of the number of publications in 2013 (n = 3), 2017 (n = 3), 2019 (n = 3), and 2020 (n = 3). These studies were conducted mainly in China (n = 6; 23.0%) and Korea (n = 5; 19.2%) followed by Italy (n = 2; 7.6%) and Japan (n = 2; 7.6%), in addition to other countries in which only 1 study was found as described in Tables 1–3.

In the 26 selected articles, 15 different species of *Citrus* were studied in a dyslipidemia model: *C. reticulata* (n = 4; 15.3%), *C. bergamia* (n = 3; 11.5%), *C. sinensis* (n = 3; 13.6%), *C. junos* Tanaka (n = 2; 9.1%), *C. grandis* (L.) Osbeck also called *C. maxima* (n = 3; 11.5%), *C. paradise* also known as grapefruit (n = 2; 7.6%), *C. unshiu* (n = 2; 7.6%), *C. sunki* Hort. Ex Tanaka (n = 1; 3.8%), *C. aurantium* (n = 1; 3.8%), *C. mitis* (n = 1; 3.8%), *C. limon* (n = 1; 3.8%), *C. aurantiifolia* (n = 1; 3.8%), *C. ichangensis* (n = 1; 3.8%), *Poncirus trifoliata* x *Citrus sinensis* (n = 1; 3.8%), and *C. changshan-huyou* (n = 1; 3.8%). Among the *Citrus* species used in the preclinical studies, there was a predominance of six hybrid species in eight studies, followed by three orange species in eight studies and three types of lemons in four publications and tangerine species in four articles. In the clinical studies, on the other hand, there is a predominance of orange-based bergamot products (*C. bergamia*; n = 3 studies) and a study with supplements containing grapefruit (*C. paradise*).

**FIGURE 2:**
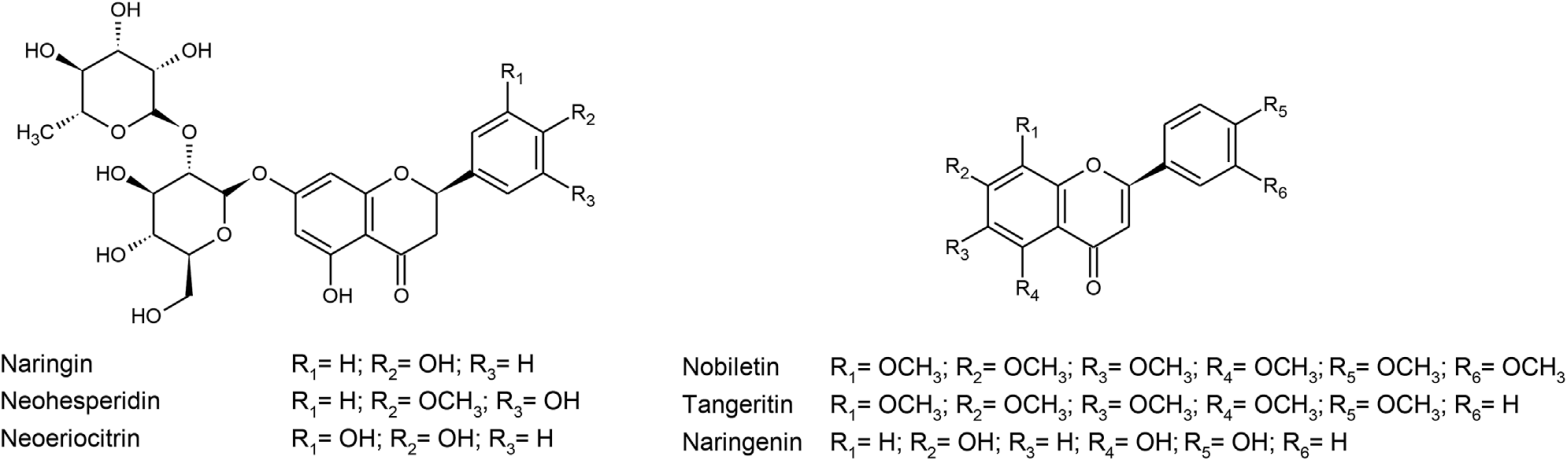
Chemical structure of the main flavonoids found in *Citrus*.

\From these species, hydroalcoholic extracts or organic fractions (n = 20; 86.9%), aqueous extract (n = 1; 4.3%), and processed fruits (n = 3; 13.0%) were used, which were incorporated to the diet (n = 14; 60.8%) or administered orally by gavage (n = 9; 40.9%). In the clinical trials as a whole, supplementation with encapsulated dry extract was used or inclusion in the diet. In addition, 21 studies (80.7%) evaluated the chemical composition of the extracts, with a predominance of compounds belonging to the class of flavonoids, such as naringin, hesperidin, neoeriocitrin, neohesperidin, nobiletin, tangeretin, and naringenin (Figure 2).

As observed in Table 1, the method of inducing hyperlipidemia in the preclinical studies was by cholesterol-rich diet or cafeteria-type diet, conducted with rats (n = 12; 52.1%), mice (n = 8; 34.7%), and hamsters (n = 3; 13.0%). Among the randomized clinical trials (Table 3), the clinical conditions of the participants were in their entirety dyslipidemia (n = 4; 100%), associated or not with coronary disease (n = 1, 25%), and hypertension and glucose intolerance (n = 1; 25%). In the preclinical and clinical studies, the outcomes evaluated were the levels of total cholesterol (TC, n = 18; 100%), HDL (n = 14; 77.7%), LDL (n = 12; 66.7%), VLDL (n = 2; 13.3%), IDL (n = 1; 5.5%), and triglycerides (TG, n = 17; 94.4%).

From the analysis of the preclinical and clinical studies (Tables 2–4), it was found that the *Citrus* species were able to significantly alter the lipid profile in the 26 (100%) studies, decreasing serum total cholesterol (n = 25; 96.1%), LDL (n = 14; 53.8%), triglycerides (n = 17; 65.3%), and VLDL (n = 2; 7.6%) and increasing HDL (n = 4; 15.3%). In the liver, *Citrus* also reduced TC and TG (n = 6; 23.0%), lipid accumulation (n = 5; 19.2%), and weight (n = 2; 7.6%). These effects were accompanied by the maintenance (n = 1; 3.8%) of glutamic-oxaloacetic transaminase (GOT), glutamic-pyruvic transaminase (GPT), and alkaline phosphatase (ALP) serum levels or the reduction of GOT, GPT (n = 2; 7.6%), and lactate dehydrogenase (LDH) (n = 1; 3.8%).

In addition, some *Citrus* products also reduced body weight gain (BWG; n = 7; 26.9%), food intake (FI; n = 1; 3.8%), and lipid accumulation in adipose tissue or cells (n = 3; 11.5%). In human, a study also demonstrated their effect on the reduction of waist circumference (WC), waist-to-hip ratio (WHR), and body mass index (BMI). Taken together, these effects can reduce the risk of atherosclerosis as shown in three studies (16.6%). However, its effects on the lipid excretion are still controversial, since two studies (11.0%) demonstrate increased excretion, two studies (11.0%) did not identify changes, and only one study (5.5%) found a reduction in excretion (Table 3). In parallel, some authors investigated the effect of *Citrus*-based products on glucose and their effects on blood glucose reduction (n = 8; 44.4%), insulin increase (n = 2; 11.0%), and glucose uptake in the cell (n = 1; 5.5%).

In addition, several targets involved in the energy and nutrient metabolism have been studied. As can be seen in Table 3, some species of *Citrus* demonstrated effects on peroxisome proliferator-activated receptor γ (PPARγ) and peroxisome proliferator-activated receptor α (PPARα), downmodulating fatty acid synthase (FAS), acyl-CoA oxidase (ACO), uncoupling protein 2 (UCP2), and adipocyte fatty-acid-binding protein (aP2), besides upregulating CD36 and acetyl-CoA carboxylase (ACC). They can also act on liver X receptor (LXR), reducing lipoprotein lipase (LPL), apolipoprotein E (ApoE), and cholesterol 7α-hydroxylase (CYP7A1) and increasing ATP-binding cassette transporter G1 (ABCG1) and ATP-binding cassette transporter A1 (ABCA1).

The adiponectin signaling pathway also can be involved in the lipid control. In fact, some *Citrus* products were able to increase adiponectin; stimulate the phosphorylation of LKB1, AMP-activated protein kinase (AMPK), ACC, and carnitine palmitoyl transferase-1 (CPT-1); and reduce HMGR and ACAT activities. Their effects on lipolysis were also observed by the upmodulation of cAMP-dependent protein kinase (PKA) and hormone-sensitive lipase (HSL), with increase in glycerol. Besides adiponectin, *Citrus* seems to act reducing other adipocytokines, as leptin and resistin, which regulate the appetite and glucose metabolism and have been associated with insulin resistance. Their effects were also observed in the hormones involved with satiety and hunger control, as leptin, glucagon-like peptide-1 (GLP-1), and ghrelin. Finally, the antioxidant potential of *Citrus* has also been demonstrated, which can offer benefits in reducing lipid oxidation and in the development of atheromatous plaques.

### Methodological Quality/Risk of Bias

The 23 preclinical studies, using the criteria provided by the ARRIVE guidelines, were analyzed for methodological quality. The studies showed a percentage of adequacy varying between 50 and 92% (83.82 ± 10.77%), with a greater weakness in the quality of the methodological description of the studies (Supplementary Table S2).

**FIGURE 3:**
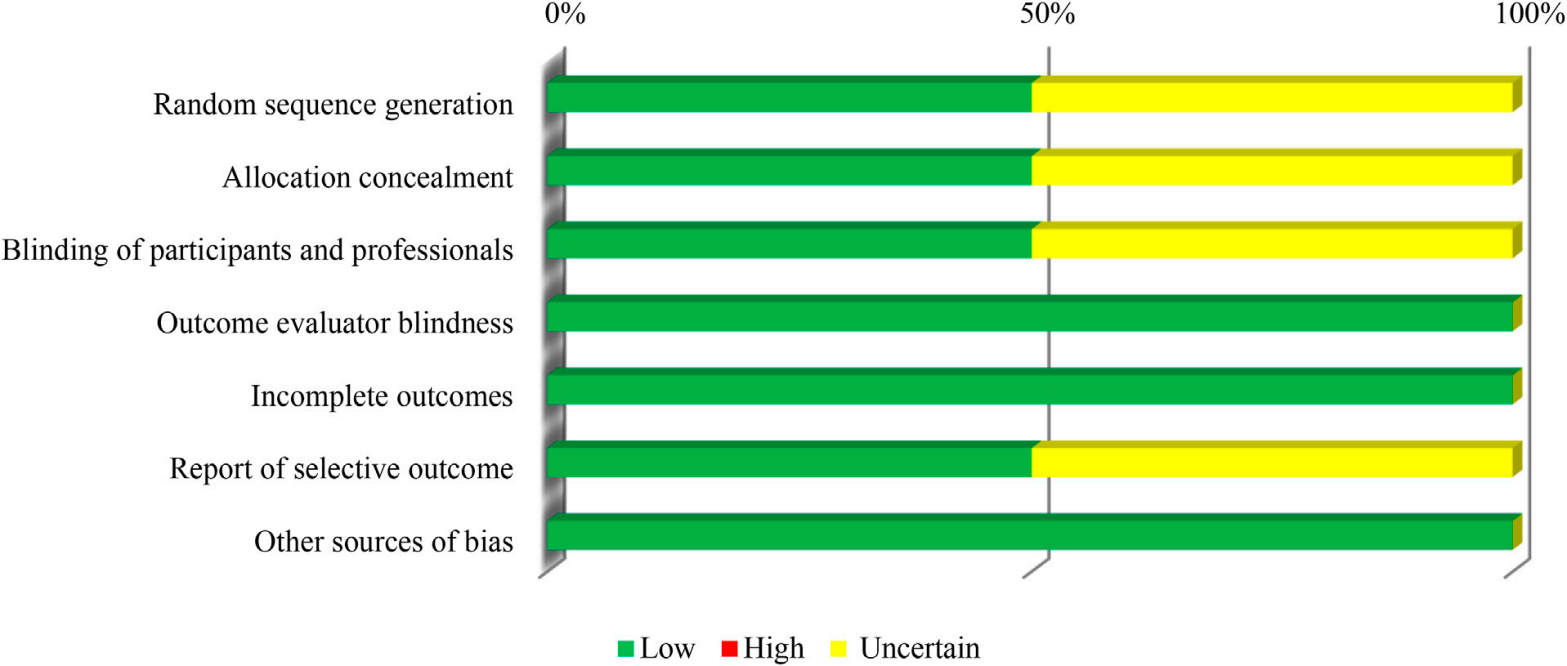
Methodological quality of clinical trials included.

As for the clinical studies included in this research and evaluated by the Cochrane list (Figure 3), all of them had blinding outcome evaluators and incomplete outcomes. In addition, 50% of the articles presented low risk of uncertain bias regarding the criteria of generating a random sequence, concealment of allocation, blinding of the participants, reporting of the selective outcome, and other sources of bias (conflict of interest, based on the source of funding for the study and method of determination of the sample size).

### Meta-Analysis

For the meta-analysis, the preclinical studies measured the level of total cholesterol [n = 23; 100%; I^2^ = 99.1% (98.9%; 99.2%)], triglycerides [n = 20; 87%; I^2^ = 99.4% (99.3%; 99.5%)], LDL [n = 12; 52.2%; I^2^ = 99.1% (98.9%; 99.3%)], and HDL [n = 14; 60.9%; I^2^ = 93.4% (90.6%; 95.4%)]. As for the clinical studies, three clinical trials with 92, 98, and 237 participants were included in the quantitative analyses, which were performed with patients with dyslipidemia and demonstrated the *Citrus* effects on the levels of total cholesterol [I^2^ = 94.5% (87.3%; 97.6%)], triglycerides [I^2^ = 95.6% (90.5%; 98.0%)], LDL [I^2^ = 96.6% (93.0%; 98.4%)], and HDL [I^2^ = 81.4% (42.2%; 94.0%)] (in both, n = 3; 100%).

**FIGURE 4:**
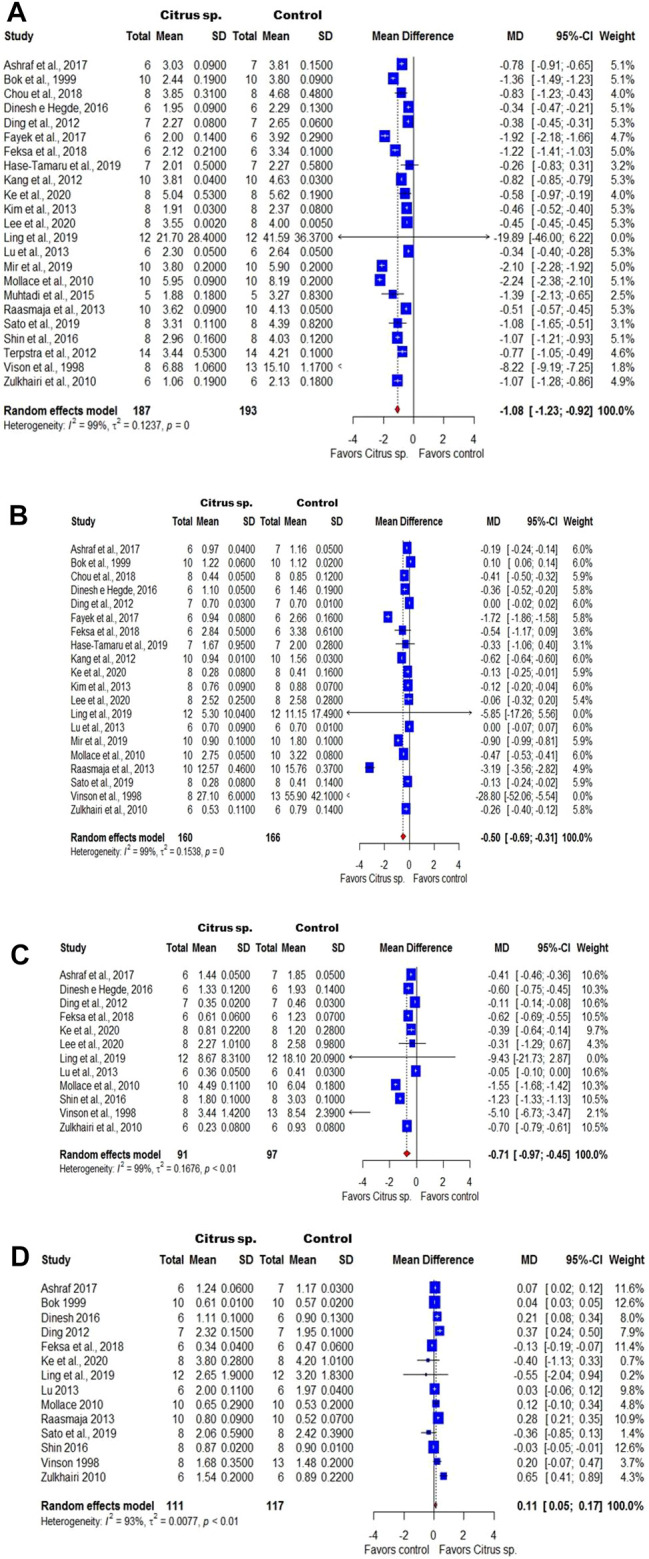
Forest plot of the preclinical studies that evaluated the effect of *Citrus* species on total cholesterol **(A)**, triglycerides **(B)**, LDL **(C)**, and HDL **(D)** levels. The numbers on the *x*-axis indicate the effect of the treatment and its favoring. SD: standard deviation of the differences. MD: difference between the means.

The presentation of the forest graphs was distributed according to the results of the levels of total cholesterol, triglycerides, LDL, and HDL for preclinical and clinical studies. Through the global analysis of preclinical studies, a reduction of −1.08 mmol/L (95% CI: 1.23; −0.92; Figure 4A) was found in total cholesterol, equivalent to 41.76 mg/dL; a reduction of −0.50 mmol/L (95% CI: 0.69; −0.31; Figure 4B) was found in triglycerides, corresponding to 44.28 mg/dL; and a reduction of −0.71 mmol/L (95% CI: 0.97; −0.45; Figure 4C) was found in LDL, what represents 27.45 mg/dL. In addition, an increase of 0.11 mmol/L in the HDL levels was verified (95% CI: 0.05; 0.17; Figure 4D), equivalent to 4.25 mg/dL.

**FIGURE 5:**
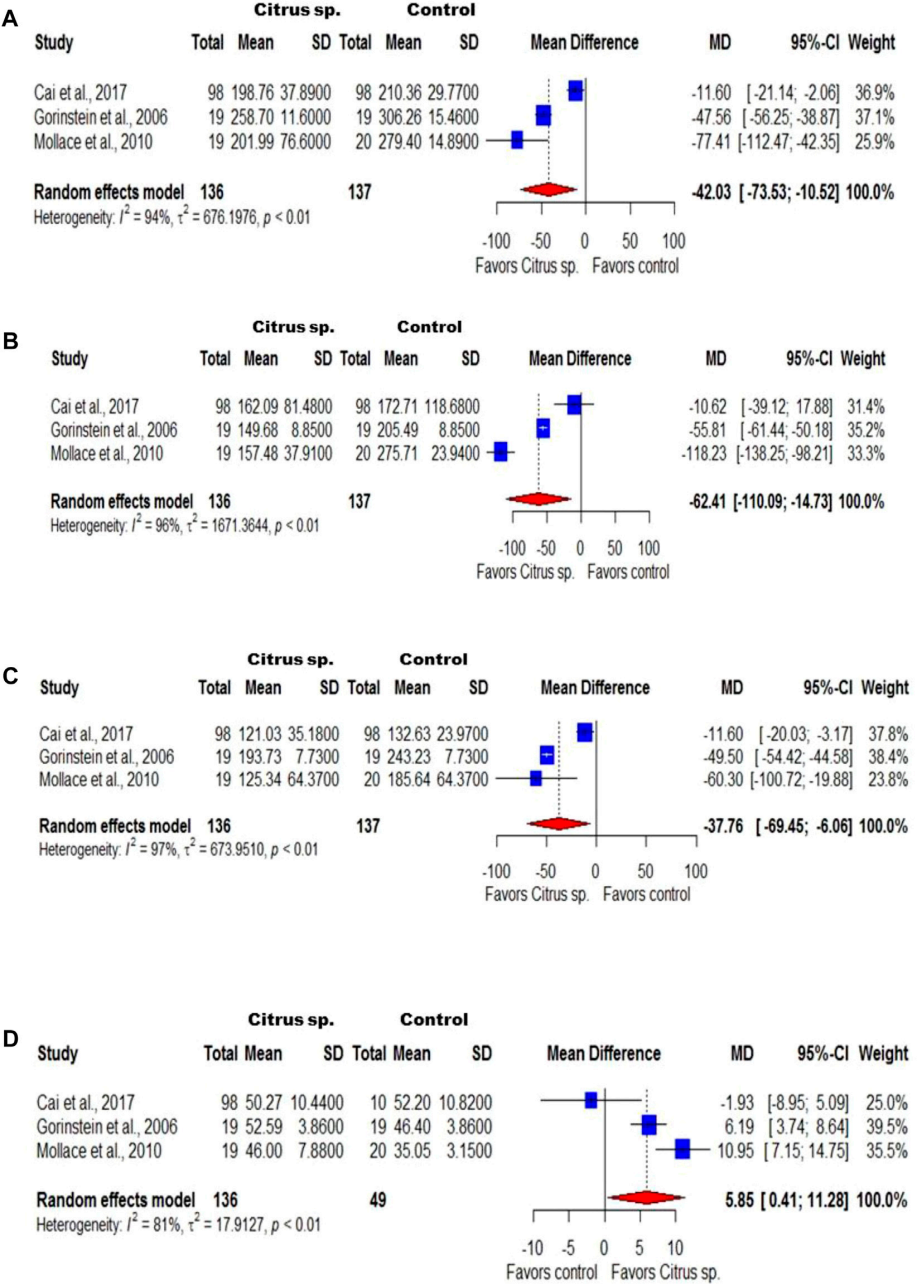
Forest plot of the clinical studies that evaluated the effect of *Citrus* species on total cholesterol **(A)**, triglyceride **(B)**, LDL **(C)**, and HDL **(D)** levels. The numbers on the *x*-axis indicate the effect of the treatment and its favoring. SD: standard deviation of the differences. MD: difference between the means.

As illustrated in Figure 5, in the studies carried out on humans, the levels (mg/dL) of total cholesterol (MD = −42.03, 95% CI: 73.53; −10.52), triglycerides (MD = −62.41, 95% CI: 110.09; −14.73), and LDL (MD = −37.76, 95% CI: 69.45; −6.06) were reduced after treating patients with *Citrus* extracts. In addition, it was observed that these patients had increased HDL levels (MD = 5.85, 95% CI: 0.41; 11.28). Although a high heterogeneity has been observed (I^2^ > 75%), the synthase of the results obtained with individual studies favors treatment to the control of serum lipids. After the analysis of subgroups, high heterogeneity was still verified and the sensitivity analysis did not change the result of the general analysis (data not shown).

## Discussion

This systematic review compiled data from 25 studies on the effects of *Citrus*-based products in the control of dyslipidemia. Based on the countries where the studies were carried out, most of them were developed in countries of Asia (such as Korea and China) and the European Union, in addition to United States and Egypt, which are among the biggest *Citrus* product makers in the world (FAS, 2018). In fact, countries that have greater production of natural resources tend to explore their products more from a commercial and scientific point of view.

Through the scientific analyses compiled, we can also verify that species of the genus *Citrus* have the potential to reduce the serum levels of total cholesterol (TC), triglycerides (TGs), LDL, and VLDL and increase HDL. Consequently, *Citrus*-based products reduced the body weight, lipid accumulation, and atherosclerosis risk by the modulation of proteins and genes involved in the lipid metabolism. Recently, a study with a standardized extract containing *Citrus sinensis* L. Osbeck associated with *Citrus limon* (Chiechio et al., 2021) also demonstrated an effect in controlling the levels of total cholesterol and triglycerides as well as glycemia, possibly due to its composition rich in anthocyanins, flavonoids, and hydroxycinnamic acids, reinstating the high potential of *Citrus* species in lipid control.

**FIGURE 6:**
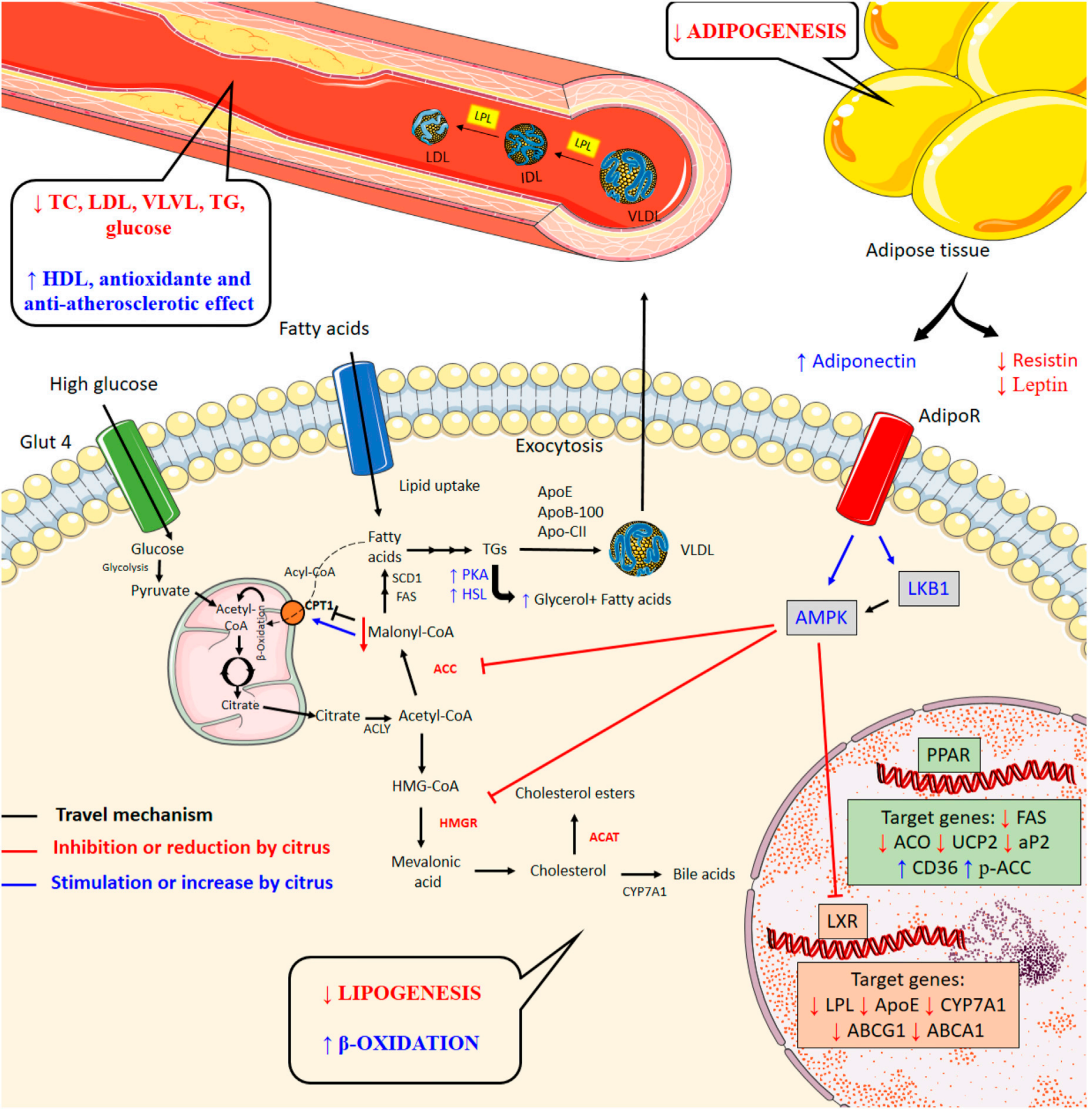
Biochemical and tissue changes caused by diets high in fat and calories (black lines) and mechanisms of action of *Citrus* products upon metabolic disorders associated with hyperlipidemia (blue lines indicate activation and red lines indicate inhibition).

These effects were studied mainly in the animal models of dyslipidemia induced by cholesterol- or high-fat diets. In these protocols, lipids ingested are initially degraded by intestinal lipase and, in enterocytes, TGs are resynthesized and associated with cholesterol and lipoproteins (ApoB-48, ApoE, and ApoC-II), forming chylomicrons. These distributed fatty acids between tissues and their remnants are metabolized in the liver. In this organ, fatty acid and glucose activate metabolic pathways for energy synthesis and storage, so that excess citrate is converted by citrate lyase (ACLY) into acetyl-CoA, which by the action of acetyl-CoA carboxylase (ACC) forms malonyl-CoA. This metabolic intermediate is used by the cell to produce fatty acid through the action of the enzymes Stearoyl-CoA Desaturase-1 (SCD1) and fatty acid synthase (FAS), in addition to downregulating CPT-1, an important transporter of Acil-Coa into the mitochondria which enables its β-oxidation. These fatty acids give rise to triglyceride molecules. In addition, acetyl-CoA can participate in the synthetic pathway of cholesterol, forming HMG-CoA which is converted into mevalonic acid by HMGR. This originates the free cholesterol molecule, which can be esterified by acyl-CoA:cholesterol acyltransferase (ACAT) or converted into bile acids by CYP7A1. TG, free cholesterol, and cholesterol ester conjugate with lipoproteins (ApoE, ApoC-II, and ApoB-100) constituting the VLDL molecule (TGs > cholesterol). This lipoprotein distributes fatty acids to tissues by the action of lipoprotein lipase (LPL) and becomes IDL (TGs ≈ cholesterol, ApoB-100, ApoE) and later LDL (TGs, < cholesterol, ApoB-100). That way, high-lipid diets increase the plasmatic concentrations of TG, TC, VLDL, IDL, and LDL (DiNicolantonio and O'Keefe, 2018; Andreadou et al., 2020). These mechanisms can be observed in Figure 6 (black lines).

Through this review, it was found that the effect of *Citrus*-based products on the release of adipocytokines and their signaling pathways has been studied. These molecules are produced by adipose tissue and control several metabolic pathways, in addition to affecting the state of hunger and satiety and being related to the development of coronary diseases and metabolic disorders (Cao, 2014). *Citrus* products reduce adiponectin (Kang et al., 2012), whose action on specific receptors (AdipoR) increases the phosphorylation of LKB1 and AMPK (Kang et al., 2012; Shin et al., 2016). It negatively modulates ACC (Kang et al., 2012; Shin et al., 2016), reducing malonyl-Coa levels and, consequently, increasing CPT-1 (Shin et al., 2016); in addition, it decreases the HMGR activity (Bok et al., 1999; Shin et al., 2016) and modulates genes like LXR (Ding et al., 2012; Lu et al., 2013) and PPAR (Kim et al., 2013; Shin et al., 2016; Lu et al., 2018). Through these genes, *Citrus* regulates several protein targets involved in lipogenesis (FAS, aP2, ACC) (Ding et al., 2012; Lu et al., 2013; Shin et al., 2016), lipoprotein formation and metabolism (ApoE, LPL) (Ding et al., 2012; Lu et al., 2013), cholesterol metabolism (CYP7A1) (Ding et al., 2012), and cholesterol and lipid efflux (ABCG1 and ABCA1) (Ding et al., 2012; Lu et al., 2013). At the same time, its ability to stimulate the PKA-HSL pathway has also been observed (Kang et al., 2012), increasing the degradation of TG in glycerol and fatty acid, in addition to reducing the activity of ACAT (Bok et al., 1999), which contributes to the reduction of cholesterol ester levels. It is worth mentioning that bio-products based on *Citrus* help in glycemic control (Mollace et al., 2011; Ding et al., 2012; Kim et al., 2013; Lu et al., 2013; Raasmaja et al., 2013; Muhtadi et al., 2015; Dinesh and Hegde, 2016; Ashraf et al., 2017; Fayek et al., 2017), possibly by reducing resistin (Kim et al., 2013), an adipocytokine whose increase has been associated with insulin resistance, atherosclerosis, oxidative stress, and inflammation. All of these molecular events result in decreased lipogenesis and increased lipid oxidation, contributing to the control of the lipid profile (Figure 6).

However, some results seem contradictory, such as the effect of *Citrus* in reduction of the mRNA levels of PPARγ target genes, including ACO and UCP2 in the liver tissue (Ding et al., 2012). ACO is the first enzyme of peroxisomal β-oxidation which will reduce the accumulation of lipids in the liver and promote its excretion (Ferdinandusse et al., 2007). On the other hand, UCP2 is an uncoupling protein which acts as a carrier of protons present in the inner membrane of mitochondria and contributes to thermogenesis, being a positive factor for the prevention of obesity (Brand and Esteves, 2005). Thus, upregulation of these mRNAs would contribute to the observed outcomes. However, the absence of baseline conditions for these targets makes it difficult to understand these data, so further studies are needed to elucidate this mechanism.

Similarly, *Citrus* seems to increase CD36 (Ding et al., 2012), the fatty acid translocase protein that facilitates the transport of fatty acids, the hepatic uptake of fatty acids, and the accumulation of fat and has a high affinity for binding with the oxidized LDL molecule, increasing the inflammatory activity and being a main condition for the development of atherosclerosis and thrombosis (Pepino et al., 2014). However, the correlation with the observed outcomes also needs to be further investigated, since the experimental conditions of the study do not allow a thorough analysis of this target in the experimental model used, as well as in the primary outcome studied.

It is also worth noting that some studies have shown that *Citrus* can help control hunger promoting the modulation of ghrelin. Known as “Hunger Hormone,” this peptide is produced by endocrine cells present in the stomach and acts in the control of hunger, adiposity, and glucose- and energy-homeostasis, among other functions (Pradhan et al., 2013). More over, *Citrus* also downregulates leptin and GLP-1 levels, which are involved with satiety control. Leptin, a hormone produced by adipose tissue, plays an important role in the control of energy homeostasis, the excess and resistance of which are associated with obesity, leading to failures in the signaling mechanisms associated with decreased nutrition and body weight control (Pan and Myers, 2018). On the other hand, glucagon-like peptide 1 (GLP-1) is a gut hormone that promotes satiety; potentiates insulin release and suppression of glucagon release in response to nutrient intake; and decreases postprandial plasma levels of glucose (Andersen et al., 2018). Thus, the effects observed for *Citrus* in the reduction of GLP-1 may be related to overnight fasting or long-term regulation of eating and energy metabolism, requiring further investigation.

The notations are as follows: ABCA1: ATP-binding cassette transporter A1; ABCG1: ATP-binding cassette transporter G1; ACAT: acyl-CoA:cholesterol acyltransferase; ACC: acetyl-CoA carboxylase; ACLY: citrate lyase; ACO: acyl-CoA oxidase; AdipoR: adiponectin receptor; AMPK: AMP-activated protein kinase; aP2: adipocyte fatty-acid-binding protein; ApoB-100: apolipoprotein B-100; ApoC-II: apolipoprotein C2; ApoE: apolipoprotein E; CD36: cluster of differentiation 36; CPT-1: carnitine palmitoyl transferase-1; CYP7A1: cholesterol 7α-hydroxylase; FAS: fatty acid synthase; GLUT 4: glucose transporter 4; HMGR: 3-hydroxy-3-methylglutaryl-coenzyme A reductase; HSL: hormone-sensitive lipase; IDL: intermediate low-density lipoprotein; LDL: low-density lipoprotein; LKB1: liver kinase B1; LPL: lipoprotein lipase; LXR: liver X receptor; p-ACC: phosphorylated acetyl-CoA carboxylase; PKA: cAMP-dependent protein kinase; PPAR: peroxisome proliferator-activated receptor; SCD1: Stearoyl-CoA Desaturase-1; TC: total cholesterol; TGs: triglycerides; UCP2: uncoupling protein 2; VLDL: very low-density lipoprotein.

The effects of *Citrus* bioproducts on the lipid profile may be related to the presence of bioactive compounds, with emphasis on the flavonoids, such as naringin, hesperidin, neohesperidin, neoeriocitrin, nobiletin, tangeretin, and naringenin as compiled in this review. In fact, these compounds are believed to play a very significant role in reducing the levels of total cholesterol, triglycerides, and LDL (Mulvihill and Huff, 2012; Assini et al., 2013; Kou et al., 2017; Zeka et al., 2017). Several studies have shown that naringin reduces the HMGR activity more potently than does vitamin E (Choi et al., 2001; Lee et al., 2001), as well as decreasing the action of ACAT (Kim et al., 2006), which contributed to hypocholesterolemic action and higher excretion of fecal sterols (Jeon et al., 2004). Similarly, hesperidin reduces plasma cholesterol in hypercholesterolemic rats by decreasing ACAT and HMGR (Lee et al., 1999; Lee et al., 2012) besides changing the expressions of genes encoding PPARs and the LDL receptor (Akiyama et al., 2009). A recent study demonstrated that neohesperidin is also able to regulate the lipid metabolism *in vivo* and *in vitro via* FGF21 and AMPK/SIRT1/PGC-1α signaling axis (Wu et al., 2017). Furthermore, the non-glycoside *Citrus* flavonoid, naringenin, stimulates the hepatic fatty acid oxidation *via* PPARγ and prevents lipogenesis in both the liver and the muscle, reducing the serum lipid levels (Mulvihill et al., 2009).

In this review, we also observed that the *Citrus* products act by reducing the atherogenic index or tissue manifestations associated with atherosclerosis (Vinson et al., 1998; Bok et al., 1999; Zulkhairi et al., 2010). In fact, the polyphenolic compounds and flavonoids found in the *Citrus* species have antioxidant (Vinson et al., 1998; Gorinstein et al., 2007; Zulkhairi et al., 2010; Craft et al., 2012) and anti-inflammatory properties, in addition to their ability to decrease LDL levels, inhibiting the formation of atherosclerotic plaques (Tripoli et al., 2007; Assini et al., 2013; Onakpoya et al., 2017). Naringin, for example, reduces plaque progression once it decreases non-high-density lipoprotein cholesterol concentrations and biomarkers of endothelial dysfunction and inhibits the expression of ICAM-1 in endothelial cells, preventing immune cell adhesion and infiltration in the vascular wall (Choe et al., 2001; Chanet et al., 2012).

Confirming the results of the systematic review, the meta-analysis of preclinical studies indicated that *Citrus* products reduce the total cholesterol, triglycerides, and LDL levels by −41.76, −44.28, and −27.45 mg/dL, respectively, while increasing the HDL levels by 4.25 mg/dL. Similar results were observed in the clinical studies, in which the *Citrus* species induce a reduction in the total cholesterol, triglycerides, and LDL levels by −42.03, −62.41, and −37.76 mg/dL, respectively, whereas the HDL levels increased by an average of 5.85 mg/dL.

In the meta-analysis published by Onakpoyaa et al. (2015) (Onakpoya et al., 2017), performed with two clinical trials about the effect of grapefruits on the lipid profile, significant effects were observed only for the increase in HDL, without TC and LDL changes. More recently, a meta-analysis published by Kou et al. (2017) showed that the sizes of effect measures for LDL and total cholesterol presented significant results in the group of patients treated with *Citrus* juice, without considerable changes in HDL and TG levels. The divergence between the results presented in our meta-analysis compared to those previously published is justified by the broader scope of our question, as well as the inclusion of more recent studies, which have confirmed the contribution of *Citrus*-based products in the control of blood lipids.

Through the analysis of the risk of bias, it can be observed that the preclinical studies have a satisfactory average score, with some limitations in the methodological description of the studies and the results. Similarly, clinical studies had limitations in reporting or methodology in terms of blinding, allocation, randomization, and reporting of results. The use of tools to assess the risk of bias in the studies included in the systematic reviews has been widely well supported by groups such as SYRCLE (Hooijmans et al., 2014), ARRIVE (Kilkenny et al., 2010), and Cochrane (Cochrane Training, 2019), since the credibility of the results and the strength of the evidence depend on the methodological criteria of the studies (Busch et al., 2020).

Thus, although the results obtained are favorable to the treatment with *Citrus* extracts, the methodological limitations and high heterogeneity of the studies included in the meta-analysis weaken the evidence about the real benefits of this intervention. In addition, the studies do not provide information on effective dose, bioavailability, efficacy, and safety. These parameters are required to propel the use of these promising therapeutic agents into the clinical area. For this reason, further studies are needed to strengthen the evidence of the effects of *Citrus* on dyslipidemia.

This systematic review presents as limitations the low evidence found due to the high variability of the studies and variation of the methodological protocols of the articles. Among them, we can mention the differences in the induction of dyslipidemia, routes of administration, and types of extracts, besides the absence of baseline serum levels of lipids for comparison after the induction and inconclusive report. Finally, as in our review, of the 25 studies included in the meta-analysis, only 3 presented results in humans; we chose not to use the GRADE system. For this reason, we believe that further clinical studies are needed to provide sufficient scientific support to measure the effectiveness of *Citrus* effects on dyslipidemia.

## Conclusion

From the compilations of the studies, one can suggest that the *Citrus* extract has a potential effect in dyslipidemia control, both in the preclinical studies and clinical trials. These effects can be associated with the presence of bioactive compounds, as flavonoids, which act synergistically through several pathways, causing inhibition of lipogenesis and activating β-oxidation. However, due to the high heterogeneity of the reposted findings, further studies are needed to increase the strength of clinical evidence of the action of *Citrus* extracts on the control of dyslipidemia and increase the strength of that evidence.

## Data Availability

The original contributions presented in the study are included in the article/Supplementary Material; further inquiries can be directed to the corresponding author.

## References

[B1] Abid H. Abid Z. Abid S. (2021). Atherogenic Indices in Clinical Practice and Biomedical Research: A Short Review. Baghdad J. Biochem. Appl. Biol. Sci. 2 (02), 60–70. 10.47419/bjbabs.v2i02.52

[B2] Adel Mehraban M. S. Tabatabaei-Malazy O. Rahimi R. Daniali M. Khashayar P. Larijani B. (2021). Targeting Dyslipidemia by Herbal Medicines: A Systematic Review of Meta-Analyses. J. Ethnopharmacology 280, 114407. 10.1016/j.jep.2021.114407 34252530

[B3] Akiyama S. Katsumata S. Suzuki K. Nakaya Y. Ishimi Y. Uehara M. (2009). Hypoglycemic and Hypolipidemic Effects of Hesperidin and Cyclodextrin-Clathrated Hesperetin in Goto-Kakizaki Rats with Type 2 Diabetes. Biosci. Biotechnol. Biochem. 73 (12), 2779–2782. 10.1271/bbb.90576 19966469

[B4] Alam M. Kauter K. Brown L. (2013). Naringin Improves Diet-Induced Cardiovascular Dysfunction and Obesity in High Carbohydrate, High Fat Diet-Fed Rats. Nutrients 5 (3), 637–650. 10.3390/nu5030637 23446977 PMC3705310

[B5] Andersen A. Lund A. Knop F. K. Vilsbøll T. (2018). Glucagon-like Peptide 1 in Health and Disease. Nat. Rev. Endocrinol. 14 (7), 390–403. 10.1038/s41574-018-0016-2 29728598

[B6] Andreadou I. Schulz R. Badimon L. Adameová A. Kleinbongard P. Lecour S. (2020). Hyperlipidaemia and Cardioprotection: Animal Models for Translational Studies. Br. J. Pharmacol. 177 (23), 5287–5311. 10.1111/bph.14931 31769007 PMC7680007

[B7] Ashraf H. Butt M. S. Iqbal M. J. Suleria H. A. R. (2017). Citrus Peel Extract and Powder Attenuate Hypercholesterolemia and Hyperglycemia Using Rodent Experimental Modeling. Asian Pac. J. Trop. Biomed. 7 (10), 870–880. 10.1016/j.apjtb.2017.09.012

[B8] Assini J. M. Mulvihill E. E. Huff M. W. (2013). Citrus Flavonoids and Lipid Metabolism. Curr. Opin. Lipidol. 24 (1), 34–40. 10.1097/MOL.0b013e32835c07fd 23254473

[B9] Atmosudigdo I. S. Lim M. A. Radi B. Henrina J. Yonas E. Vania R. (2021). Dyslipidemia Increases the Risk of Severe COVID-19: A Systematic Review, Meta-analysis, and Meta-regression. Clin. Med. Insights : Endocrinol. Diabetes 4, 1–7. 10.1177/1179551421990675 PMC8842489 35173508

[B10] Ballard C. R. Galvão T. F. Cazarin C. B. B. Maróstica M. R. (2019). Effects of Polyphenol-Rich Fruit Extracts on Diet-Induced Obesity in Rodents: Systematic Review and Meta-Analysis. Curr. Pharm. Des. 25 (32), 3484–3497. 10.2174/1381612824666191010170100 31608836

[B11] Bok S. H. Lee S. H. Park Y. B. Bae K. H. Son K. H. Jeong T. S. (1999). Plasma and Hepatic Cholesterol and Hepatic Activities of 3-Hydroxy-3-Methyl-Glutaryl-CoA Reductase and Acyl CoA: Cholesterol Transferase Are Lower in Rats Fed Citrus Peel Extract or a Mixture of Citrus Bioflavonoids. J. Nutr. 129 (6), 1182–1185. 10.1093/jn/129.6.1182 10356084

[B12] Brand M. D. Esteves T. C. (2005). Physiological Functions of the Mitochondrial Uncoupling Proteins UCP2 and UCP3. Cell Metab 2 (2), 85–93. 10.1016/j.cmet.2005.06.002 16098826

[B13] Busch L. M. Sun J. Cui X. Eichacker P. Q. Torabi-Parizi P. (2020). Checkpoint Inhibitor Therapy in Preclinical Sepsis Models: a Systematic Review and Meta-Analysis. Intensive Care Med. Exp. 8 (1), 7. 10.1186/s40635-019-0290-x 32020483 PMC7000606

[B14] Cai Y. Xing G. Shen T. Zhang S. Rao J. Shi R. (2017). Effects of 12-week Supplementation of Citrus Bergamia Extracts-Based Formulation CitriCholess on Cholesterol and Body Weight in Older Adults with Dyslipidemia: a Randomized, Double-Blind, Placebo-Controlled Trial. Lipids Health Dis. 16 (1), 251. 10.1186/s12944-017-0640-1 29273027 PMC5741859

[B15] Cao H. (2014). Adipocytokines in Obesity and Metabolic Disease. J. Endocrinol. 220 (2), T47–T59. 10.1530/JOE-13-0339 24403378 PMC3887367

[B16] Chanet A. Wizinska P. Polakof S. Mazur A. Bennetau-Pelissero C. Morand C. (2012). Naringin at a Nutritional Dose Modulates Expression of Genes Related to Lipid Metabolism and Inflammation in Liver of Mice Fed a High-Fat Diet. Nutr. Aging 1 (2), 113–123. 10.3233/nua-2012-0010

[B17] Chiechio S. Zammataro M. Barresi M. Amenta M. Ballistreri G. Fabroni S. (2021). A Standardized Extract Prepared from Red Orange and Lemon Wastes Blocks High-Fat Diet-Induced Hyperglycemia and Hyperlipidemia in Mice. Molecules 26 (14), 4291. 10.3390/molecules26144291 34299566 PMC8304280

[B18] Choe S. C. Kim H. S. Jeong T. S. Bok S. H. Park Y. B. (2001). Naringin Has an Antiatherogenic Effect with the Inhibition of Intercellular Adhesion Molecule-1 in Hypercholesterolemic Rabbits. J. Cardiovasc. Pharmacol. 38 (6), 947–955. 10.1097/00005344-200112000-00017 11707699

[B19] Choi M. S. Do K. M. Park Y. S. Jeon S. M. Jeong T. S. Lee Y. K. (2001). Effect of Naringin Supplementation on Cholesterol Metabolism and Antioxidant Status in Rats Fed High Cholesterol with Different Levels of Vitamin E. Ann. Nutr. Metab. 45 (5), 193–201. 10.1159/000046729 11585976

[B20] Chou Y. C. Ho C. T. Pan M. H. (2018). Immature Citrus Reticulata Extract Promotes Browning of Beige Adipocytes in High-Fat Diet-Induced C57BL/6 Mice. J. Agric. Food Chem. 66 (37), 9697–9703. 10.1021/acs.jafc.8b02719 30146891

[B21] Cochrane Training (2019). Cochrane Handbook for Systematic Reviews of Interventions. Wiley Online Books. Available at: : https://onlinelibrary.wiley.com/doi/book/10.1002/9781119536604.

[B22] Craft B. D. Kerrihard A. L. Amarowicz R. Pegg R. B. (2012). Phenol-Based Antioxidants and the *In Vitro* Methods Used for Their Assessment. Compr. Rev. Food Sci. Food Saf. 11 (2), 148–173. 10.1111/j.1541-4337.2011.00173.x

[B23] Dinesh S. S. Hegde K. (2016). Antiobesity Activity of Ethanolic Extract ofCitrus Maximaleaves on Cafeteria Diet Induced and Drug Induced Obese Rats. Rese. Jour. Pharm. Technol. 9 (7), 907–912. 10.5958/0974-360x.2016.00173.6

[B24] Ding X. Fan S. Lu Y. Zhang Y. Gu M. Zhang L. (2012). Citrus Ichangensis Peel Extract Exhibits Anti-metabolic Disorder Effects by the Inhibition of PPARγ and LXR Signaling in High-Fat Diet-Induced C57BL/6 Mouse. Evid. Based Complement. Alternat Med. 2012, 678592. 10.1155/2012/678592 23320036 PMC3536358

[B25] DiNicolantonio J. J. O'Keefe J. H. (2018). Effects of Dietary Fats on Blood Lipids: a Review of Direct Comparison Trials. Open Heart 5 (2), e000871. 10.1136/openhrt-2018-000871 30094038 PMC6074619

[B26] FAS (2018). Citrus: World Markets and Trade. Washington: USDA Foreign Agricultural Service. Available at: https://www.fas.usda.gov/data/citrus-world-markets-and-trade.

[B27] Fayek N. M. El-Shazly A. H. Abdel-Monem A. R. Moussa M. Y. Abd-Elwahab S. M. El-Tanbouly N. D. (2017). Comparative Study of the Hypocholesterolemic, Antidiabetic Effects of Four Agro-Waste Citrus Peels Cultivars and Their HPLC Standardization. Revista Brasileira de Farmacognosia 27 (4), 488–494. 10.1016/j.bjp.2017.01.010

[B28] Feksa D. L. Coelho R. P. Aparecida da Costa Güllich A. Dal Ponte E. S. da Costa Escobar Piccoli J. Manfredini V. (2018). Extract of Citrus Maxima (Pummelo) Leaves Improve Hepatoprotective Activity in Wistar Rats Submitted to the Induction of Non-alcoholic Hepatic Steatosis. Biomed. Pharmacother. 98, 338–346. 10.1016/j.biopha.2017.12.070 29274591

[B29] Ferdinandusse S. Denis S. Hogenhout E. M. Koster J. van Roermund C. W. IJlst L. (2007). Clinical, Biochemical, and Mutational Spectrum of Peroxisomal Acyl-Coenzyme A Oxidase Deficiency. Hum. Mutat. 28 (9), 904–912. 10.1002/humu.20535 17458872

[B30] Fruchart J. C. Sacks F. M. Hermans M. P. Assmann G. Brown W. V. Ceska R. (2008). The Residual Risk Reduction Initiative: a Call to Action to Reduce Residual Vascular Risk in Dyslipidaemic Patient. Diab Vasc. Dis. Res. 5 (4), 319–335. 10.3132/dvdr.2008.046 18958843

[B31] Gattuso G. Barreca D. Gargiulli C. Leuzzi U. Caristi C. (2007). Flavonoid Composition of Citrus Juices. Molecules 12 (8), 1641–1673. 10.3390/12081641 17960080 PMC6149096

[B32] Geleijnse J. M. Launer L. J. Hofman A. Pols H. A. Witteman J. C. (1999). Tea Flavonoids May Protect against Atherosclerosis: the Rotterdam Study. Arch. Intern. Med. 159 (18), 2170–2174. 10.1001/archinte.159.18.2170 10527294

[B33] Gorinstein S. Leontowicz H. Leontowicz M. Krzeminski R. Gralak M. Jastrzebski Z. (2007). Effect of Hesperidin and Naringin on the Plasma Lipid Profile and Plasma Antioxidant Activity in Rats Fed a Cholesterol-Containing Diet. J. Sci. Food Agric. 87 (7), 1257–1262. 10.1002/jsfa.2834

[B34] Hase-Tamaru S. Okushima A. Miyata Y. Nakayama H. Aramaki S. Miyata Y. (2019). Unripe and Discarded Satsuma Mandarin (Citrus Unshiu MARC.) Improves Lipid Metabolism in Rats. Fstr 25, 705–713. 10.3136/fstr.25.705

[B35] Higgins J. P. Thompson S. G. (2002). Quantifying Heterogeneity in a Meta-Analysis. Stat. Med. 21 (11), 1539–1558. 10.1002/sim.1186 12111919

[B36] Hooijmans C. R. Rovers M. M. de Vries R. B. Leenaars M. Ritskes-Hoitinga M. Langendam M. W. (2014). SYRCLE's Risk of Bias Tool for Animal Studies. BMC Med. Res. Methodol. 14, 43. 10.1186/1471-2288-14-43 24667063 PMC4230647

[B37] Ingersgaard M. V. Helms Andersen T. Norgaard O. Grabowski D. Olesen K. (2020). Reasons for Nonadherence to Statins - A Systematic Review of Reviews. Patient Prefer Adherence 14, 675–691. 10.2147/PPA.S245365 32308373 PMC7135196

[B38] Jeon S. M. Park Y. B. Choi M. S. (2004). Antihypercholesterolemic Property of Naringin Alters Plasma and Tissue Lipids, Cholesterol-Regulating Enzymes, Fecal Sterol and Tissue Morphology in Rabbits. Clin. Nutr. 23 (5), 1025–1034. 10.1016/j.clnu.2004.01.006 15380892

[B39] Kang S. I. Shin H. S. Kim H. M. Hong Y. S. Yoon S. A. Kang S. W. (2012). Immature Citrus Sunki Peel Extract Exhibits Antiobesity Effects by β-oxidation and Lipolysis in High-Fat Diet-Induced Obese Mice. Biol. Pharm. Bull. 35 (2), 223–230. 10.1248/bpb.35.223 22293353

[B40] Ke Z. Zhao Y. Tan S. Chen H. Li Y. Zhou Z. (2020). Citrus Reticulata Blanco Peel Extract Ameliorates Hepatic Steatosis, Oxidative Stress and Inflammation in HF and MCD Diet-Induced NASH C57BL/6 J Mice. J. Nutr. Biochem. 83, 108426. 10.1016/j.jnutbio.2020.108426 32559586

[B41] Khan W. Augustine D. Rao R. S. Patil S. Awan K. H. Sowmya S. V. (2021). Lipid Metabolism in Cancer: A Systematic Review. J. Carcinog 20, 4. 10.4103/jcar.JCar_15_20 34321955 PMC8312377

[B42] Kilkenny C. Browne W. J. Cuthill I. C. Emerson M. Altman D. G. (2010). Improving Bioscience Research Reporting: The ARRIVE Guidelines for Reporting Animal Research. Plos Biol. 8 (6), e1000412. 10.1371/journal.pbio.1000412 20613859 PMC2893951

[B43] Kim S. H. Hur H. J. Yang H. J. Kim H. J. Kim M. J. Park J. H. (2013). Citrus Junos Tanaka Peel Extract Exerts Antidiabetic Effects via AMPK and PPAR-γ Both *In Vitro* and *In Vivo* in Mice Fed a High-Fat Diet. Evid. Based Complement. Alternat Med. 2013, 921012. 10.1155/2013/921012 23762167 PMC3674686

[B44] Kim S. Y. Kim H. J. Lee M. K. Jeon S. M. Do G. M. Kwon E. Y. (2006). Naringin Time-Dependently Lowers Hepatic Cholesterol Biosynthesis and Plasma Cholesterol in Rats Fed High-Fat and High-Cholesterol Diet. J. Med. Food 9 (4), 582–586. 10.1089/jmf.2006.9.582 17201649

[B45] Kou G. Zhao Z. Dong X. Zhang Y. Guo L. Zhou Z. (2017). Effects of Citrus Fruits on Blood Lipid Levels: A Systematic Review and Meta- Analysis. Acta Med. Mediterr. 33, 1143–1150. 10.19193/0393-6384_2017_6_179

[B46] Lamiquiz-Moneo I. Giné-González J. Alisente S. Bea A. M. Pérez-Calahorra S. Marco-Benedí V. (2020). Effect of Bergamot on Lipid Profile in Humans: A Systematic Review. Crit. Rev. Food Sci. Nutr. 60 (18), 3133–3143. 10.1080/10408398.2019.1677554 31670973

[B47] Lee C. H. Jeong T. S. Choi Y. K. Hyun B. H. Oh G. T. Kim E. H. (2001). Anti-atherogenic Effect of Citrus Flavonoids, Naringin and Naringenin, Associated with Hepatic ACAT and Aortic VCAM-1 and MCP-1 in High Cholesterol-Fed Rabbits. Biochem. Biophys. Res. Commun. 284 (3), 681–688. 10.1006/bbrc.2001.5001 11396955

[B48] Lee G. H. Peng C. Park S. A. Hoang T. H. Lee H. Y. Kim J. (2020). Citrus Peel Extract Ameliorates High-Fat Diet-Induced NAFLD via Activation of AMPK Signaling. Nutrients 12 (3), 673. 10.3390/nu12030673 PMC7146518 32121602

[B49] Lee S.-H. Jeong T.-S. Park Y. B. Kwon Y.-K. Choi M.-S. Bok S.-H. (1999). Hypocholesterolemic Effect of Hesperetin Mediated by Inhibition of 3-Hydroxy-3-Methylgultaryl Coenzyme a Reductase and Acyl Coenzyme a: Cholesterol Acyltransferase in Rats Fed High-Cholesterol Diet. Nutr. Res. 19 (8), 1245–1258. 10.1016/s0271-5317(99)00085-8

[B50] Lee Y. S. Huh J. Y. Nam S. H. Moon S. K. Lee S. B. (2012). Enzymatic Bioconversion of Citrus Hesperidin by Aspergillus Sojae Naringinase: Enhanced Solubility of Hesperetin-7-O-Glucoside with *In Vitro* Inhibition of Human Intestinal Maltase, HMG-CoA Reductase, and Growth of Helicobacter pylori. Food Chem. 135 (4), 2253–2259. 10.1016/j.foodchem.2012.07.007 22980799

[B51] Ling Y. Shi Z. Yang X. Cai Z. Wang L. Wu X. (2020). Hypolipidemic Effect of Pure Total Flavonoids from Peel of Citrus (PTFC) on Hamsters of Hyperlipidemia and its Potential Mechanism. Exp. Gerontol. 130, 110786. 10.1016/j.exger.2019.110786 31760082

[B52] Lu M. Cao Y. Xiao J. Song M. Ho C. T. (2018). Molecular Mechanisms of the Anti-obesity Effect of Bioactive Ingredients in Common Spices: a Review. Food Funct. 9 (9), 4569–4581. 10.1039/c8fo01349g 30168574

[B53] Lu Y. Xi W. Ding X. Fan S. Zhang Y. Jiang D. (2013). Citrange Fruit Extracts Alleviate Obesity-Associated Metabolic Disorder in High-Fat Diet-Induced Obese C57BL/6 Mouse. Ijms 14 (12), 23736–23750. 10.3390/ijms141223736 24317433 PMC3876074

[B54] Mach F. Baigent C. Catapano A. L. Koskinas K. C. Casula M. Badimon L. (2020). 2019 ESC/EAS Guidelines for the Management of Dyslipidaemias: Lipid Modification to Reduce Cardiovascular Risk. Eur. Heart J. 41 (1), 111–188. 10.1093/eurheartj/ehz455 31504418

[B55] Mir H. Krouf D. Taleb-Dida N. Berzou S. Guenzet A. Khelladi H. (2019). Effects of Citrus Latifolia Extract on Dyslipidemia and Tissues Redox Status in Rats Fed a High-Cholesterol Diet. Nfs 49 (6), 989–999. 10.1108/nfs-04-2018-0110

[B56] Mollace V. Sacco I. Janda E. Malara C. Ventrice D. Colica C. (2011). Hypolipemic and Hypoglycaemic Activity of Bergamot Polyphenols: from Animal Models to Human Studies. Fitoterapia 82 (3), 309–316. 10.1016/j.fitote.2010.10.014 21056640

[B57] Muhtadi M. Haryoto H. Azizah T. Suhendi A. Yen K. (2015). Antidiabetic and Antihypercholesterolemic Activities of Citrus Sinensis Peel: *In Vivo* Study. Natl. J. Physiol. Pharm. Pharmacol. 5 (5), 382–385. 10.5455/njppp.2015.5.2807201561

[B58] Mulvihill E. E. Allister E. M. Sutherland B. G. Telford D. E. Sawyez C. G. Edwards J. Y. (2009). Naringenin Prevents Dyslipidemia, Apolipoprotein B Overproduction, and Hyperinsulinemia in LDL Receptor-Null Mice with Diet-Induced Insulin Resistance. Diabetes 58 (10), 2198–2210. 10.2337/db09-0634 19592617 PMC2750228

[B59] Mulvihill E. E. Huff M. W. (2012). Protection from Metabolic Dysregulation, Obesity, and Atherosclerosis by Citrus Flavonoids: Activation of Hepatic PGC1α-Mediated Fatty Acid Oxidation. PPAR Res. 2012, 857142. 10.1155/2012/857142 22701469 PMC3369495

[B60] Onakpoya I. O'Sullivan J. Heneghan C. Thompson M. (2017). The Effect of Grapefruits (Citrus Paradisi) on Body Weight and Cardiovascular Risk Factors: A Systematic Review and Meta-Analysis of Randomized Clinical Trials. Crit. Rev. Food Sci. Nutr. 57 (3), 602–612. 10.1080/10408398.2014.901292 25880021

[B61] Page M. J. McKenzie J. E. Bossuyt P. M. Boutron I. Hoffmann T. C. Mulrow C. D. (2021). The PRISMA 2020 Statement: an Updated Guideline for Reporting Systematic Reviews. BMJ 372, n71. 10.1136/bmj.n71 33782057 PMC8005924

[B62] Pan W. W. Myers M. G. (2018). Leptin and the Maintenance of Elevated Body Weight. Nat. Rev. Neurosci. 19 (2), 95–105. 10.1038/nrn.2017.168 29321684

[B63] Patil V. C. Avhad A. B. Kulkarni A. R. Pandere K. A. (2020). High-Sensitive C-Reactive Protein in Patients with Coronary Artery Disease. J. Nat. Sci. Biol. Med. 11, 39–44. 10.4103/jnsbm.JNSBM_159_19

[B64] Pepino M. Y. Kuda O. Samovski D. Abumrad N. A. (2014). Structure-Function of CD36 and Importance of Fatty Acid Signal Transduction in Fat Metabolism. Annu. Rev. Nutr. 34, 281–303. 10.1146/annurev-nutr-071812-161220 24850384 PMC4329921

[B65] Pirillo A. Casula M. Olmastroni E. Norata G. D. Catapano A. L. (2021). Global Epidemiology of Dyslipidaemias. Nat. Rev. Cardiol. 18 (10), 689–700. 10.1038/s41569-021-00541-4 33833450

[B66] Pradhan G. Samson S. L. Sun Y. (2013). Ghrelin: Much More Than a Hunger Hormone. Curr. Opin. Clin. Nutr. Metab. Care 16 (6), 619–624. 10.1097/MCO.0b013e328365b9be 24100676 PMC4049314

[B67] Raasmaja A. Lecklin A. Li X. M. Zou J. Zhu G. G. Laakso I. (2013). A Water-Alcohol Extract of Citrus Grandis Whole Fruits Has Beneficial Metabolic Effects in the Obese Zucker Rats Fed with High Fat/high Cholesterol Diet. Food Chem. 138 (2–3), 1392–1399. 10.1016/j.foodchem.2012.09.140 23411259

[B68] Rafiq S. Kaul R. Sofi S. A. Bashir N. Nazir F. Ahmad Nayik G. (2018). Citrus Peel as a Source of Functional Ingredient: A Review. J. Saudi Soc. Agric. Sci. 17, 351. Available at: http://www.sciencedirect.com/science/article/pii/S1658077X16300960. 10.1016/j.jssas.2016.07.006

[B69] Sahebkar A. (2017). Effects of Quercetin Supplementation on Lipid Profile: A Systematic Review and Meta-Analysis of Randomized Controlled Trials. Crit. Rev. Food Sci. Nutr. 57 (4), 666–676. 10.1080/10408398.2014.948609 25897620

[B70] Sato M. Goto T. Inoue E. Miyaguchi Y. Toyoda A. (2019). Dietary Intake of Immature Citrus Tumida Hort. Ex Tanaka Peels Suppressed Body Weight Gain and Fat Accumulation in a Mouse Model of Acute Obesity. J. Nutr. Sci. Vitaminol (Tokyo) 65 (1), 19–23. 10.3177/jnsv.65.19 30814407

[B71] Schulz I. (2006). Treatment of Dyslipidemia: How and when to Combine Lipid Lowering Drugs. Arq Bras Endocrinol. Metabol 50 (2), 344–359. 10.1590/s0004-27302006000200021 16767301

[B72] Shin E. J. Park J. H. Sung M. J. Chung M. Y. Hwang J. T. (2016). Citrus Junos Tanaka Peel Ameliorates Hepatic Lipid Accumulation in HepG2 Cells and in Mice Fed a High-Cholesterol Diet. BMC Complement. Altern. Med. 16 (1), 499. 10.1186/s12906-016-1460-y 27912736 PMC5135759

[B73] Sowndarya K. Joseph J. Shenoy A. Hegde A. (2021). Evaluation of Triglyceride/high-Density Lipoprotein Ratio as a Surrogate Marker for Insulin Resistance in Healthy Young Males. J. Nat. Sci. Biol. Med. 12 (2), 213–217. 10.4103/jnsbm.JNSBM-193-20

[B74] Manuel T Marco C Fred G (Editors) (2020). 1st Edition. Available at: https://www.elsevier.com/books/the-genus-citrus/talon/978-0-12-812163-4.The Genus Citrus

[B75] Terpstra A. H. Lapré J. A. de Vries H. T. Beynen A. C. (2002). The Hypocholesterolemic Effect of Lemon Peels, Lemon Pectin, and the Waste Stream Material of Lemon Peels in Hybrid F1B Hamsters. Eur. J. Nutr. 41 (1), 19–26. 10.1007/s003940200002 11990004

[B76] Toth P. P. Patti A. M. Nikolic D. Giglio R. V. Castellino G. Biancucci T. (2015). Bergamot Reduces Plasma Lipids, Atherogenic Small Dense LDL, and Subclinical Atherosclerosis in Subjects with Moderate Hypercholesterolemia: A 6 Months Prospective Study. Front. Pharmacol. 6, 299. 10.3389/fphar.2015.00299 26779019 PMC4702027

[B77] Tripoli E. Guardia M. L. Giammanco S. Majo D. D. Giammanco M. (2007). Citrus Flavonoids: Molecular Structure, Biological Activity and Nutritional Properties: A Review. Food Chem. 104 (2), 466–479. 10.1016/j.foodchem.2006.11.054

[B78] Vinson J. A. Hu S.-J. Jung S. Stanski A. M. (1998). A Citrus Extract Plus Ascorbic Acid Decreases Lipids, Lipid Peroxides, Lipoprotein Oxidative Susceptibility, and Atherosclerosis in Hypercholesterolemic Hamsters. J. Agric. Food Chem. 46 (4), 1453–1459. 10.1021/jf970801u

[B79] Wiggins B. S. Dixon D. Bellone J. Gasbarro N. Marrs J. C. Tran R. (2019). Key Articles and Guidelines in the Management of Dyslipidemia: 2019 Update. J. Pharm. Pract. 11, 0897190019868413. 10.1177/0897190019868413 31401932

[B80] Wu H. Liu Y. Chen X. Zhu D. Ma J. Yan Y. (2017). Neohesperidin Exerts Lipid-Regulating Effects *In Vitro* and *In Vivo* via Fibroblast Growth Factor 21 and AMP-Activated Protein Kinase/Sirtuin Type 1/Peroxisome Proliferator-Activated Receptor Gamma Coactivator 1α Signaling Axis. Pharmacology 100 (3–4), 115–126. 10.1159/000452492 28554169

[B81] Zeka K. Ruparelia K. Arroo R. R. J. Budriesi R. Micucci M. (2017). Flavonoids and Their Metabolites: Prevention in Cardiovascular Diseases and Diabetes. Diseases 5 (3), 19. 10.3390/diseases5030019 PMC5622335 32962323

[B82] Zhao X. Wang D. Qin L. (2021). Lipid Profile and Prognosis in Patients with Coronary Heart Disease: a Meta-Analysis of Prospective Cohort Studies. BMC Cardiovasc. Disord. 21 (1), 69. 10.1186/s12872-020-01835-0 33535982 PMC7860615

[B83] Zulkhairi H. A. Khairunnuur A. F. Hafipah M. R. N. Azrina A. Rasadah M. A. Kamilah K. A. K. (2010). An Aqueous Extract of Citrus Mitis Possesses Antioxidative Properties and Improves Plasma Lipid Profiles in Rat Induced with High Cholesterol Diet. J. Med. Plant Res. 4 (1), 49–57. 10.5897/JMPR09.385

