# Supplementary material for: Citrus Extract as a Perspective for the Control of Dyslipidemia: A Systematic Review With Meta-Analysis From Animal Models to Human Studies
Source: Front Pharmacol. 2022 Feb 14;13:822678. doi: 10.3389/fphar.2022.822678 (PMC8884359; doi:10.3389/fphar.2022.822678)
Supplement: Supplementary file 1 [file DataSheet1.docx]

Supplementary Material

**Supplementary Table 1 –** Search strategies used in Pubmed, Scopus, Scielo, Lilacs databases.

| Data base | Search strategy |
| --- | --- |
| Pubmed | "CITRUS"[Mesh] AND "Lipoproteins"[Mesh] OR "Cholesterol"[Mesh] OR "Dyslipidemias"[Mesh] OR "Hypercholesterolemia"[Mesh] OR "Hyperlipidemias"[Mesh] |
| Scopus | "CITRUS" AND "Lipoproteins" OR "Cholesterol" OR "Epicholesterol" OR "Dyslipidemias" OR "Dyslipoproteinemia" OR "Hypercholesterolemia" OR "High Cholesterol Levels" OR "Hyperlipidemias" OR "Lipidemia", |
| Scielo | "CITRUS" AND "Lipoproteins" OR "Cholesterol" OR "Epicholesterol" OR "Dyslipidemias" OR "Dyslipoproteinemia" OR "Hypercholesterolemia" OR "High Cholesterol Levels" OR "Hyperlipidemias" OR "Lipidemia" |
| Lilacs | "CITRUS" AND "Lipoproteins" OR "Cholesterol" OR "Epicholesterol" OR "Dyslipidemias" OR "Dyslipoproteinemia" OR "Hypercholesterolemia" OR "High Cholesterol Levels" OR "Hyperlipidemias" OR "Lipidemia" |

**Supplementary Table 2 – Assessment of the methodological quality of the pre-clinical studies through the ARRIVE guide**.

|  | **Quality of Studies** | |  |  |  |  |  |  |  |  |  |  |  |  |  |  |  |  |  |  |  |  |  |  |  |
| --- | --- | --- | --- | --- | --- | --- | --- | --- | --- | --- | --- | --- | --- | --- | --- | --- | --- | --- | --- | --- | --- | --- | --- | --- | --- |
| **1** | **Title** | Vinson et al., 1998 | Ashraf et al., 2017 | Mollace et al., 2010 | Muhtadi et al., 2015 | Terpstra et al., 2002 | Ding et al., 2012 | Bok et al., 1999 | Raasmaja et al., 2013 | Lu et al., 2013 | Zulkhairi et al., 2010 | Dinesh; Hegde, 2016 | Kang et al.,2012 | Shin et al., 2016 | Kim et al., 2013 | Fayek et al., 2017 | Chou et al., 2018 | Feksa et al., 2018 | Mir et al., 2019 | Sato et al., 2019 | Tamaru et al., 2019 | Lee et al., 2020 | Ling et al., 2020 | Ke et al., 2020 | **(%)** |
|  | Accurate and concise description of article content | X | X | X | X | X | X | - | X | X | X | X | X | X | X | X | X | X | X | X | X | X | X | X | 95 |
| **2** | **Abstract** |  |  |  |  |  |  |  |  |  |  |  |  |  |  |  |  |  |  |  |  |  |  |  |  |
|  | Summary of background, research objectives, methods, main findings and conclusions | X | X | X | X | X | X | X | X | X | X | X | X | X | X | X | X | X | X | X | X | X | X | X | 100 |
| **3** | **Introduction** |  |  |  |  |  |  |  |  |  |  |  |  |  |  |  |  |  |  |  |  |  |  |  |  |
|  | Sufficient scientific background | X | X | X | - | X | X | - | X | X | X | X | X | X | X | X | X | X | X | X | X | X | X | X | 91 |
|  | Explanation of the methodological approach, species and studied parts | X | X | X | - | X | X | X | X | X | X | X | X | X | X | X | X | X | X | X | X | X | X | X | 95 |
| **4** | **Objectives** |  |  |  |  |  |  |  |  |  |  |  |  |  |  |  |  |  |  |  |  |  |  |  |  |
|  | Clear primary and secondary objectives |  | X | X | X | X | X | X | X | X | X | X | X | X | X | X | X | X | X | X | X | X | X | X | 95 |
| **5** | **Materials and methods** |  |  |  |  |  |  |  |  |  |  |  |  |  |  |  |  |  |  |  |  |  |  |  |  |
| **5.1** | **Ethical problems** |  |  |  |  |  |  |  |  |  |  |  |  |  |  |  |  |  |  |  |  |  |  |  |  |
|  | Nature of ethical review permits, relevant permits and national or institutional guidelines for the care and use of animals | X | X | X | X | X | X | X | X | - | X | X | X | X | X | X | X | X | X | X | X | X | X | X | 95 |
| **6** | **Study design** |  |  |  |  |  |  |  |  |  |  |  |  |  |  |  |  |  |  |  |  |  |  |  |  |
|  | Number of animals per group (either per analysis or per species) | X | - | X | X | X | X | X | X | - | X | X | X | X | X | X | X | X | X | X | X | X | X | X | 91 |
| **7** | **Experimental procedures** |  |  |  |  |  |  |  |  |  |  |  |  |  |  |  |  |  |  |  |  |  |  |  |  |
|  | Description of the techniques used | X | X | X | X | X | X | X | X | - | X | X | X | X | X | X | X | X | X | X | X | X | X | X | 95 |
|  | Number of portions analyzed | X | - | X | X | X | X | X | X | - | X | X | X | X | X | X | X | X | X | X | X | X | X | X | 91 |
| **8** | **Experimental animals** |  |  |  |  |  |  |  |  |  |  |  |  |  |  |  |  |  |  |  |  |  |  |  |  |
|  | Information about animals | X | - | X | X | X | X | X | X | X | X | X | X | X | X | X | X | X | X | X | X | X | X | X | 95 |
|  | Location of animal origin (place of collection) | X | X | X | X | X | X | X | X |  | X | X | X | X | X | X | X | X | X | X | X | X | X | X | 95 |
|  | Animal sex | X | - | X | X | X | X | X | X | - | X | X | X | X | X | X | X | X | X | X | X | X | X | X | 91 |
|  | Size (length) of animals (Average) | X | - | X | X | X | X | X | X | - | X | X | X | - | X | X | X | X | X | X | X | X | X | X | 87 |
|  | Animal weight range | X | X | X | - | X | X | X | X | X | X | X | X | - | X | X | X | X | X | X | X | X | X | X | 91 |
|  | Animal age | X | - | - | - | X | X | X | X | - | X | - | X | X | X | X | - | X | X | X | X | X | X | X | 73 |
| **9** | **Accommodation and breeding** |  |  |  |  |  |  |  |  |  |  |  |  |  |  |  |  |  |  |  |  |  |  |  |  |
|  | Housing of experimental animals (type of facility, type of cage or housing, material, number of cage mates) | X | - | X | - | - | - | - | X | - | X | X | X | X | - | X | X | X | X | X | X | X | X | X | 69 |
|  | Breeding conditions (breeding program, light/dark cycle, temperature, water, food) | X | X | X | - | - | X | X | X | - | X | X | X | X | X | X | X | X | X | X | X | X | X | X | 87 |
| **10** | **Sample size** |  |  |  |  |  |  |  |  |  |  |  |  |  |  |  |  |  |  |  |  |  |  |  |  |
|  | Number of animals for analysis / technique | X | - | X | X | X | X | X | X | - | X | X | X | X | X | X | X | X | X | X | X | X | X | X | 91 |
|  | Explanation for this number | - | - | - | - | - | - | - | - | - | - | - | - | - | - | - | - | - | - | - | - | - | - | - | 0 |
| **11** | **Statistical methods** |  |  |  |  |  |  |  |  |  |  |  |  |  |  |  |  |  |  |  |  |  |  |  |  |
|  | Specification of analyzed parameters | X | X | X | X | X | X | X | X | X | X | X | X | X | X | X | X | X | X | X | X | X | X | X | 100 |
|  | Statistical methods used for each analysis | X | X | X | X | X | X | X | X | X | X | X | X | X | X | X | X | X | X | X | X | X | X | X | 100 |
|  | Methods used to assess whether the data meet the premises of the statistical approach | - | - | - | - | - | - | - | - | - | - | - | - | - | - | - | - | - | - | - | - | - | - | - | 0 |
| **12** | **Results** |  |  |  |  |  |  |  |  |  |  |  |  |  |  |  |  |  |  |  |  |  |  |  |  |
| **12.1** | **Results and Estimate** |  |  |  |  |  |  |  |  |  |  |  |  |  |  |  |  |  |  |  |  |  |  |  |  |
|  | Description of results (text quality, tables, graphs, figures. | X | X | X | X | X | X | X | X | X | X | X | X | X | X | X | X | X | X | X | X | X | X | X | 100 |
|  | Statistical information (means ± standard deviation) | X | - | X | X | X | - | - | - | - | - | - | X | X | X | X | X | X | X | X | X | X | X | X | 69 |
| **13** | **Discussion** |  |  |  |  |  |  |  |  |  |  |  |  |  |  |  |  |  |  |  |  |  |  |  |  |
| **13.1** | **Scientific interpretation / implications** |  |  |  |  |  |  |  |  |  |  |  |  |  |  |  |  |  |  |  |  |  |  |  |  |
|  | Interpretation of results, taking into account the objectives and hypotheses of the study, current theory and relevant studies | X | X | X | X | X | X | X | X | X | X | X | X | X | X | X | X | X | X | X | X | X | X | X | 100 |
|  | Comments on study limitations (inaccuracy associated with results) | X | X | X | X | X | X | X | X | X | X | X | X | X | X | X | X | X | X | X | X | X | X | X | 100 |
| **14** | **Generalization / translation** |  |  |  |  |  |  |  |  |  |  |  |  |  |  |  |  |  |  |  |  |  |  |  |  |
|  | Comments on functional and evolutionary aspects | X | X | X | X | X | X | X | X | X | X | X | X | X | X | X | X | X | X | X | X | X | X | X | 100 |
| **15** | **Financing** |  |  |  |  |  |  |  |  |  |  |  |  |  |  |  |  |  |  |  |  |  |  |  |  |
|  | List of funding sources and the role of funders) in the study | - | - | - | X | X | X | X | - | X | X | - | X | X | X | - | X | X | - | X | X | - | - | - | 56 |
|  | **Total result (%)** | 85 | 57 | 85 | 71 | 85 | 85 | 78 | 85 | 50 | 89 | 82 | 92 | 85 | 89 | 89 | 89 | 92 | 89 | 92 | 92 | 89 | 89 | 89 | **Average**  **83.82%** |

**Supplementary Table 3:** SMILES and InChL keys of the molecules reported in the study.

| Molecules | InChI Key | Canonical SMILES |
| --- | --- | --- |
| Naringin | DFPMSGMNTNDNHN-ZPHOTFPESA-N | CC1C(C(C(C(O1)OC2C(C(C(OC2OC3=CC(=C4C(=O)CC(OC4=C3)C5=CC=C(C=C5)O)O)CO)O)O)O)O)O |
| Neohesperidin | ARGKVCXINMKCAZ-UHFFFAOYSA-N | CC1C(C(C(C(O1)OC2C(C(C(OC2OC3=CC(=C4C(=O)CC(OC4=C3)C5=CC(=C(C=C5)OC)O)O)CO)O)O)O)O)O |
| Neoeriocitrin | OBKKEZLIABHSGY-DOYQYKRZSA-N | CC1C(C(C(C(O1)OC2C(C(C(OC2OC3=CC(=C4C(=O)CC(OC4=C3)C5=CC(=C(C=C5)O)O)O)CO)O)O)O)O)O |
| Nobiletin | MRIAQLRQZPPODS-UHFFFAOYSA-N | COC1=C(C=C(C=C1)C2=CC(=O)C3=C(O2)C(=C(C(=C3OC)OC)OC)OC)OC |
| Tangeritin | ULSUXBXHSYSGDT-UHFFFAOYSA-N | COC1=CC=C(C=C1)C2=CC(=O)C3=C(O2)C(=C(C(=C3OC)OC)OC)OC |
| Naringenin | FTVWIRXFELQLPI-UHFFFAOYSA-N | C1C(OC2=CC(=CC(=C2C1=O)O)O)C3=CC=C(C=C3)O |
